# Machine learning-based risk prediction of acute kidney disease and hospital mortality in older patients

**DOI:** 10.3389/fmed.2024.1407354

**Published:** 2024-08-15

**Authors:** Xinyuan Wang, Lingyu Xu, Chen Guan, Daojun Xu, Lin Che, Yanfei Wang, Xiaofei Man, Chenyu Li, Yan Xu

**Affiliations:** ^1^Department of Nephrology, The Affiliated Hospital of Qingdao University, Qingdao, China; ^2^Department of Nephrology, Linyi People's Hospital, Linyi, China

**Keywords:** acute kidney disease, hospital mortality, risk prediction, machine learning, older people

## Abstract

**Introduction:**

Acute kidney injury (AKI) is a prevalent complication in older people, elevating the risks of acute kidney disease (AKD) and mortality. AKD reflects the adverse events developing after AKI. We aimed to develop and validate machine learning models for predicting the occurrence of AKD, AKI and mortality in older patients.

**Methods:**

We retrospectively reviewed the medical records of older patients (aged 65 years and above). To explore the trajectory of kidney dysfunction, patients were categorized into four groups: no kidney disease, AKI recovery, AKD without AKI, or AKD with AKI. We developed eight machine learning models to predict AKD, AKI, and mortality. The best-performing model was identified based on the area under the receiver operating characteristic curve (AUC) and interpreted using the Shapley additive explanations (SHAP) method.

**Results:**

A total of 22,005 patients were finally included in our study. Among them, 4,434 patients (20.15%) developed AKD, 4,000 (18.18%) occurred AKI, and 866 (3.94%) patients deceased. Light gradient boosting machine (LGBM) outperformed in predicting AKD, AKI, and mortality, and the final lite models with 15 features had AUC values of 0.760, 0.767, and 0.927, respectively. The SHAP method revealed that AKI stage, albumin, lactate dehydrogenase, aspirin and coronary heart disease were the top 5 predictors of AKD. An online prediction website for AKD and mortality was developed based on the final models.

**Discussion:**

The LGBM models provide a valuable tool for early prediction of AKD, AKI, and mortality in older patients, facilitating timely interventions. This study highlights the potential of machine learning in improving older adult care, with the developed online tool offering practical utility for healthcare professionals. Further research should aim at external validation and integration of these models into clinical practice.

## 1 Introduction

Acute kidney injury (AKI), a complex public health concern, prevalent in about 12% of patients (1–3) and often accompanied by multiple organ failure especially in older people, which leads to up to 1.7 million annual deaths (4–8). Studies reported that older AKI survivors face a considerable risk of progressing to chronic kidney disease (CKD) (9). The poor prognosis of older patients with kidney disease poses significant challenges to the healthcare system and result in a substantial economic burden on families due to multi-system damage or long-term hemodialysis treatment.

Current evidence indicates that AKI can progress to an intermediate stage called acute kidney disease (AKD), defined by the 16th Acute Disease Quality Initiative (ADQI) meeting as acute or subacute damage and/or loss of kidney function for 7–90 days after an AKI-initiating event (9). Distinguishing between AKD and AKI in clinical practice is crucial, as the management strategies and prognostic implications for these conditions differ. While AKI represents a sudden decline in kidney function, AKD encompasses a broader timeframe and includes patients who do not fully recover from an episode of AKI, presenting a poorer prognosis in older patients, with a study showing a 31.8% in-hospital mortality rate for older patients in the validation cohorts (10). Explaining this clinical distinction is vital for understanding the progression of kidney diseases and the necessity for targeted prediction models. As a transitional period, AKD may serve as a turning point for improving patients’ renal function and presents significant potential for clinical research. Developing accurate prediction models for AKD has substantial clinical implications. These models can facilitate early identification of at-risk patients, enabling timely interventions that may prevent further kidney damage and improve patient outcomes. However, current studies mainly focus on AKI, with insufficient exploration of AKD’s impacts and trajectories in the older adult, underscoring the importance of targeted research on AKD.

Recently, several studies have demonstrated that the superior predictive capabilities of machine learning (ML) models over traditional statistical methods in predicting AKI. For instance, in pediatric critical care, the prediction of Stage 2/3 AKI by a ML model showed an AUROC of 0.89 (11). The random forest (RF) model for predicting AKI in patients undergoing cardiac surgery achieved an AUC of 0.839 (12). Despite ML’s complexity, the SHapley Additive exPlanation (SHAP) method has been developed to make these models more interpretable (13, 14). Nevertheless, the application of ML and SHAP methods for the prediction of AKD in older patients remains limited.

Hence, the primary aim of this study was to investigate the incidence rates of AKD, AKI, and mortality among older patients, addressing a gap in the epidemiology of kidney injury trajectories in the older adult. Secondly, we aimed to pioneer the development of predictive ML models for AKD, AKI, and mortality. Furthermore, we integrated the SHAP approach to bolster the interpretability of prediction models. Finally, we have also developed an innovative online risk calculator rooted in ML algorithms. These may provide a critical window for early targeted interventions to improve the prognosis of the older adult, thereby alleviating pressure on healthcare systems.

## 2 Materials and methods

### 2.1 Data collection

We retrospectively reviewed the medical records of 40,325 patients aged ≥65 years between October 2012 and October 2019. Patients were excluded if they met one of the following criteria: continuous dialysis, renal transplantation before AKD diagnosis, less than two serum creatinine (Scr) tests during hospitalization or missing inpatient data and the duration of hospitalization <48 h. We collected data on demographic characteristics, comorbidities, laboratory parameters, and medications from the hospital information system. Comorbidities mentioned in this study were all defined according to the International Classification of Disease (ICD) 10th Revision. The study was approved by the Institutional Review Board (IRB; QYFY WZLL 28250), ensuring patient confidentiality through anonymized data collection and adherence to privacy protocols.

### 2.2 Definition

The primary outcome was the occurrence of AKD, with secondary outcomes including AKI and mortality. AKI was diagnosed based on Kidney Disease: Improving Global Outcomes (KDIGO) 2012 as follows: Scr level > 26.5 mmol/L (0.3 mg/dL) within 48 h; an increase in Scr to more than 1.5-fold the baseline-confirmed value or an increase presumed to have occurred within 7 days; or urine output <0.5 mL/kg/h for more than 6 h (15). AKD was defined following the 2017 ADQI as acute or subacute damage and/or loss of kidney function for a duration of between 7 and 90 days after exposure to an AKI initiating event (9). Diagnosis and staging of AKI and AKD were determined at the first fulfillment of these criteria.

Based on the diagnostic criteria of AKI and AKD, patients were classified into the following four groups. AKI Recovery: This group included patients whose Scr levels returned to baseline within 7 days, indicating a renal impairment duration of less than 7 days or a rapid recovery within that timeframe. AKD without AKI: This group comprised patients whose Scr levels increased gradually but remained elevated for more than 7 days, indicating subacute AKD without meeting the AKI criteria. AKD with AKI: Patients in this category experienced stage ≥1 AKI that persisted for at least 7 days after the initial AKI event, indicating a continuous progression from AKI to AKD. No Kidney Disease (NKD): Patients falling into this category had an eGFR of 60 mL/min/1.73 m^2^ or higher, no detectable albuminuria, and did not meet the criteria for either AKI or AKD. To thoroughly assess the influence of evolving kidney injury patterns on mortality among older patients, we integrated AKI and AKD into a unified metric termed ‘dynamic’ during the mortality model’s construction. The ‘dynamic’ variable adopts values 0, 1, 2 and 3 corresponding to NKD, AKI recovery, AKD without AKI, and AKD with AKI, respectively. Baseline Scr was defined as the first Scr value measured during hospitalization. The baseline estimated glomerular filtration rate (eGFR) was calculated using the Chronic Kidney Disease Epidemiology Collaboration formula (16).

### 2.3 Model development

We engineered predictive models for AKD, AKI, and mortality, respectively. Scikit-learn (https://github.com/scikit-learn/scikitlearn) package was used to build models including logistic regression (LR), support vector machine (SVM), random forest (RF), naïve byes (NB), k-nearest neighbor (KNN), multi-layer perceptron (MLP), gradient boosting machine (GBM) and light gradient boosting machine (LGBM). The data were divided, with 80% utilized for training and 20% for testing. Grid search method with ten-fold cross validation was used in the training set to prevent overfitting and to identify the optimal hyperparameters for each model. To address the disparity in the distribution of positive and negative samples, we implemented a strategy of class weight adjustment during the training phase of the ML model (17).

### 2.4 Model interpretation and evaluation

SHAP method was designed to address the “black-box” issue in prediction models by providing a means to rank the importance of input features and explain model results (14, 18). This approach offers both global and local explanations, enhancing our understanding of the model’s decision-making process. Globally, it provides consistent attribution values for each feature, revealing associations. Locally, it explains specific predictions for individual cases, enhancing interpretability. In our pursuit of feature optimization, we also utilized the SHAP method for feature selection in the optimal model. SHAP value-assisted feature selection was utilized to identify the top 20, 15, 10, and 5 features for model construction. This approach was to find the best balance between accuracy and complexity, leading to a final lite model. SHAP method was implemented using Python shap package (https://shap.readthedocs.io/en/latest/).

The performance of our predictive models was evaluated on the test set, focusing on their discriminative ability and clinical utility. Discrimination was quantitatively assessed using a suite of performance metrics, including area under curve (AUC) of the receiver operating characteristic (ROC) curve (19), sensitivity, specificity, recall, accuracy, F1 score, Brier score and Matthews correlation coefficient (MCC). The model demonstrating the highest AUC was designated as the optimal one. For clinical applicability, decision curve analysis (DCA) was employed, which calculated the net benefit of the final model by contrasting the predicted benefits against the expected risks associated with the outcomes (20). Furthermore, the performance of the final model was showed through precision-recall (PR) curves, Kolmogorov–Smirnov (KS) plots, and confusion matrix.

### 2.5 Online prediction website

We created an online web-based risk calculator utilizing the Streamlit Python framework, employing the model with the optimal number of features. Upon the values of corresponding features are provided, the website can return the probability of AKD and mortality, respectively. This tool showed the practical application of our research in a clinical setting.

### 2.6 Sensitivity analysis

A sensitivity analysis was performed to thoroughly examine the predictive efficacy of the models, focusing specifically on stages 2–3 of AKD. Additionally, the models’ performance underwent a thorough assessment across various subgroups, with a particular emphasis on patients stratified by age brackets: 65–74 years, 75–84 years, and those aged over 85 years.

### 2.7 Statistical analysis

Variables with over 15% missing values were excluded, while those with less than 15% missing data were imputed using the Multivariate Imputation by Chained Equations (MICE) algorithm (21). Continuous variables were shown as mean with standard deviation, or median with interquartile range and compared by the Independent-sample T test or Wilcoxon rank-sum test. Categorial variables were expressed in quantities and percentages and compared by the Chi-square tests. All analyses were carried out with Python version 3.10.11, R version 4.3.1, and SPSS version 25.0. A 2-tailed *p* value of <0.05 was considered statistically significant.

## 3 Results

### 3.1 Patient characteristics

In total, this study enrolled 22,005 patients (Supplementary Figure S1), in which 4,434 patients (20.15%) developed AKD, and 4,000 (18.18%) occurred AKI. Specifically, 2,237 patients (10.17%) had AKD with AKI, 2,671 (12.14%) had AKD without AKI, and 1,763 (8.01%) had AKI recovery. On top of that, there were 866 (3.94%) patients deceased. In the AKD group, 3,553 patients (16.15%) were at stage 1, 663 (3.01%) at stage 2, and 218 (0.99%) at stage 3. These findings suggested that the high occurrence of AKI and AKD among older patients.

The differences in characteristics between kidney injury group and NKD group are partially shown in Table 1, with a detailed comparison of all characteristics provided in Supplementary Table S1. In brief, compared to the NKD group, patients with acute/subacute kidney dysfunction were older on average (75.00 ± 13.00 vs. 73.00 ± 12.00, *p* < 0.05) with more risk factors like smoking, alcohol use, diabetes and other conditions. The baseline lab tests including eGFR, blood urea nitrogen (BUN), cystatin C (Cys), blood glucose, lipid profiles, uric acid (UA) and others were also worst in kidney dysfunction group (*p* < 0.05). Furthermore, the data indicated that patients with renal impairment endured longer hospital stays (18.00 ± 14.00 vs. 17.00 ± 9.00 days, p < 0.05) and encountered higher hospital mortality rates (9.6% vs. 1.5%, p < 0.05) in comparison to the NKD group. This signified that older patients with kidney dysfunction were susceptible to a worsening prognosis.

**Table 1:** The partial baseline characteristics of the current cohort.

Variables	NKD (n = 15,334)	Acute/subacute renal impairment					
		AKI recovery (*n* = 2,237)	AKD without AKI (*n* = 2,671)	AKD with AKI (*n* = 1763)	Total (*N* = 6,671)	|*t*/*Z*/*χ*2|	*p*-value
**Demographics**							
Age (years)	73.00 (12.00)	75.00 (13.00)	76.00 (14.00)	75.00 (12.00)	75.00 (13.00)	11.731	<0.001
Male, *n* (%)	9,474 (61.80)	1,308 (58.50)	1,473 (55.10)	1,023 (58.00)	3,804 (57.00)	44.038	<0.001
BMI (kg/m^2^)	23.71 (5.06)	24.00 (45.30)	23.44 (5.19)	23.66 (5.34)	23.66 (5.22)	1.884	0.060
SBP (mmHg)	132.00 (28.00)	133.00 (30.00)	131.00 (28.00)	130.00 (29.00)	131.00 (29.00)	2.536	0.011
DBP (mmHg)	78.00 (14.00)	78.00 (17.00)	75.00 (15.00)	76.00 (18.00)	76.00 (17.00)	4.808	<0.001
Smoke, *n* (%)	5,738 (37.40)	741 (33.10)	856 (32.00)	574 (32.60)	2,171 (32.50)	48.009	<0.001
Drink, *n* (%)	4,227 (27.60)	532 (23.80)	635 (23.80)	440 (25.00)	1,607 (24.10)	28.843	<0.001
**Laboratory data**							
Scr (umol/L)	83.00 (27.92)	82.00 (59.00)	82.00 (41.00)	83.00 (69.80)	82.00 (52.73)	1.835	0.066
eGFR (ml/min/1.73^2^)	70.96 (20.42)	68.36 (32.93)	68.75 (26.45)	67.77 (33.69)	68.36 (30.72)	6.322	<0.001
BUN (mmol/L)	5.78 (2.89)	6.70 (5.59)	6.14 (4.49)	6.92 (6.42)	6.52 (5.22)	19.940	<0.001
Cys (mg/L)	1.00 (0.46)	1.08 (0.87)	1.09 (0.69)	1.23 (1.13)	1.12 (0.82)	21.540	<0.001
Glucose (mmol/L)	5.53 (2.15)	6.50 (3.90)	5.92 (2.79)	6.50 (3.78)	6.24 (3.42)	22.283	<0.001
CK (U/L)	66.00 (64.48)	73.00 (93.05)	55.00 (70.00)	71.00 (108.00)	65.00 (88.40)	0.392	0.695
Hb (g/L)	122.02 (23.48)	118.94 (27.07)	112.81 (25.21)	112.75 (26.46)	114.85 (26.33)	19.161	<0.001
PLT (10^9^/L)	225.98 (95.49)	207.07 (89.12)	217.94 (102.33)	204.51 (95.89)	210.74 (96.54)	10.793	<0.001
RBC (10^12^/L)	4.08 (0.71)	3.96 (0.87)	3.80 (0.80)	3.77 (0.86)	3.85 (0.84)	19.962	<0.001
WBC (10^9^/L)	6.42 (3.17)	7.42 (5.09)	7.06 (4.18)	7.50 (5.10)	7.33 (4.73)	21.405	<0.001
ALT (U/L)	17.00 (15.00)	19.00 (23.00)	18.00 (20.00)	21.00 (32.00)	19.00 (23.50)	11.193	<0.001
GGT (U/L)	20.00 (25.00)	21.40 (36.65)	25.00 (41.00)	28.00 (63.70)	25.00 (43.00)	15.013	<0.001
TBIL (umol/L)	13.40 (9.30)	14.20 (12.68)	13.90 (12.50)	15.80 (17.07)	14.50 (13.46)	10.485	<0.001
AST (U/L)	18.00 (11.00)	20.00 (22.00)	20.00 (17.00)	23.00 (29.00)	20.50 (21.00)	18.597	<0.001
HDL (mmol/L)	1.23 (0.41)	1.15 (0.48)	1.14 (0.46)	1.08 (0.55)	1.13 (0.49)	14.933	<0.001
LDH (U/L)	163.00 (60.00)	179.00 (93.00)	181.00 (93.00)	193.00 (105.00)	183.60 (96.00)	25.053	<0.001
UA (umol/L)	291.82 (114.30)	323.06 (161.04)	293.09 (145.84)	318.84 (175.47)	309.55 (159.83)	8.192	<0.001
LDL (mmol/L)	2.61 (1.25)	2.38 (1.30)	2.35 (1.30)	2.28 (1.35)	2.34 (1.31)	16.083	<0.001
A/G (mmol/L)	1.26 (0.34)	1.22 (0.43)	1.15 (0.33)	1.17 (0.33)	1.18 (0.37)	16.356	<0.001
ALB (g/L)	35.09 (6.07)	33.19 (6.53)	32.07 (6.32)	32.07 (6.54)	32.44 (6.47)	28.366	<0.001
PT (s)	10.50 (2.20)	11.30 (3.00)	11.10 (2.50)	11.40 (3.00)	11.20 (2.80)	26.097	<0.001
FIB (g/L)	3.53 (1.09)	3.56 (1.19)	3.62 (1.20)	3.59 (1.24)	3.59 (1.21)	3.544	<0.001
TT (s)	14.50 (3.20)	14.90 (3.60)	15.00 (3.60)	15.00 (3.60)	15.00 (3.60)	11.825	<0.001
**Comorbidities, *n* (%)**							
CKD	630 (4.10)	181 (8.10)	210 (7.90)	249 (14.10)	640 (9.60)	257.189	<0.001
Respiratory failure	514 (3.40)	128 (5.70)	235 (8.80)	172 (9.80)	535 (8.00)	223.101	<0.001
Diabetes	3,296 (21.50)	469 (21.00)	688 (25.80)	448 (25.40)	1,605 (24.10)	17.662	<0.001
Atrial fibrillation	221 (1.40)	69 (3.10)	134 (5.00)	103 (5.80)	306 (4.60)	196.797	<0.001
CHD	4,153 (27.10)	662 (29.60)	1,052 (39.40)	619 (35.10)	2,333 (35.00)	139.166	<0.001
Shock	87 (0.60)	77 (3.40)	48 (1.80)	140 (7.90)	265 (4.00)	342.416	<0.001
Hypertension	6,611 (43.10)	919 (41.10)	1,328 (49.70)	807 (45.80)	3,054 (45.80)	13.423	<0.001
**Medications, *n* (%)**							
*β*-receptor blocker	5,539 (36.10)	791 (35.40)	962 (36.00)	810 (45.90)	2,563 (38.40)	10.550	0.001
ACEI	1,242 (8.10)	249 (11.10)	390 (14.60)	247 (14.00)	886 (13.30)	142.887	<0.001
ARB	3,059 (19.90)	418 (18.70)	661 (24.70)	372 (21.10)	1,451 (21.80)	9.261	0.002
β-lactam antibiotics	5,629 (36.70)	920 (41.10)	1,276 (47.80)	978 (55.50)	3,174 (47.60)	228.844	<0.001
Cardiac glycosides	8,209 (53.50)	1,371 (61.30)	1,502 (56.20)	1,270 (72.00)	4,143 (62.10)	138.653	<0.001
Aspirin	5,461 (35.6)	929 (41.5)	1,201 (45.0)	933 (52.9)	3,063 (45.9)	202.865	<0.001
Omeprazole	6,396 (41.70)	796 (35.60)	1,254 (46.90)	868 (49.20)	2,918 (43.70)	7.850	0.005
**Outcomes**							
Hospital mortality, n (%)	223 (1.50)	167 (7.50)	140 (5.20)	336 (19.10)	643 (9.60)	823.653	<0.001
LOS (days)	17.00 (9.00)	14.00 (11.00)	20.00 (12.00)	20.00 (16.00)	18.00 (14.00)	2.691	0.007

Values are presented as mean with standard deviation, or median with interquartile range unless stated otherwise. The P-value was calculated among NKD and three subtypes of acute/subacute renal impairment. * BMI: body mass index; SBP: systolic blood pressure; DBP: diastolic blood pressure; Scr: serum creatinine; eGFR: estimated glomerular filtration rate; BUN: blood urea nitrogen; Cys, cystatin C; CK: creatine kinase; Hb: hemoglobin; PLT: platelet; RBC: red blood cell; WBC: white blood cell; ALT: alanine aminotransferase; GGT: gamma glutamyl transferase; TBIL: total bilirubin; AST: aspartate aminotransferase; HDL: high-density lipoprotein; LDH: lactate dehydrogenase; UA: uric acid; LDL: low-density lipoprotein; A/G: albumin/globulin ratio; ALB: albumin; PT: prothrombin time; FIB: fibrinogen; TT: thrombin time; CKD: chronic kidney disease; ACEI: angiotensin-converting enzyme inhibitors; ARB: angiotensin II receptor blockers; CCB: calcium channel blockers; CHD: coronary heart disease; LOS: length of stay.

### 3.2 Feature selection and model performance

**Figure 1:**
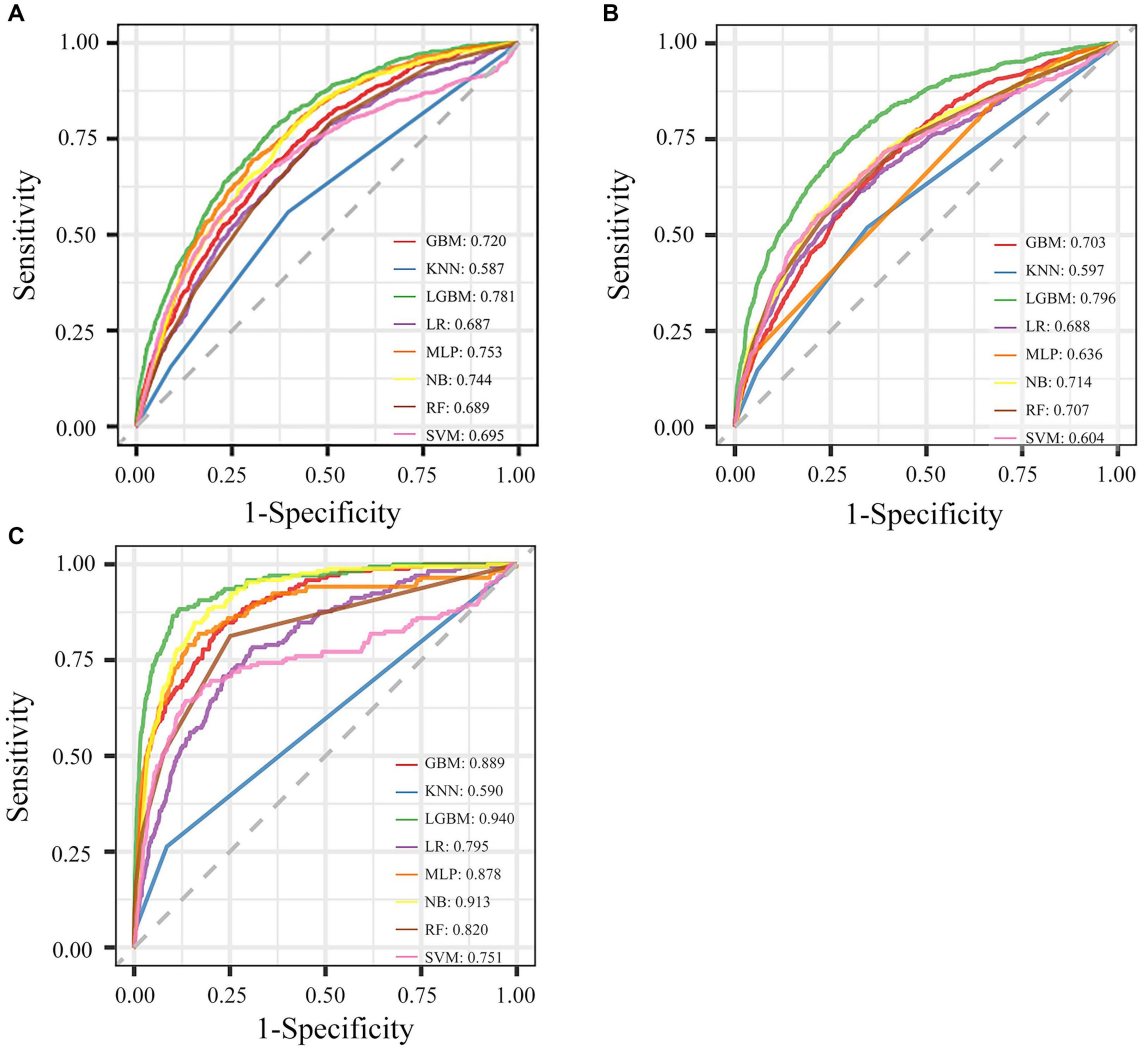
Performance of eight ML models for different outcomes with all features. **(A)** The ROC curve of AKD. **(B)** The ROC curve of AKI. **(C)** The ROC curve of mortality.

Eight ML models were developed to predict AKD occurrence in older patients, by utilizing all available features, with the ROC curves illustrated in Figure 1A. The LGBM model emerged as the most efficacious in predicting AKD, achieving an AUC of 0.781. The performance metrics of these eight ML models in predicting AKD were comprehensively tabulated in Table 2. Given LGBM’s superior performance, we subsequently conducted a feature selection process specifically within the LGBM model framework. Additionally, Supplementary Figure S2 presented a correlation matrix heatmap, delineating the interrelationships between the predictive outcomes of the various ML models.

**Table 2:** Performance of eight ML models for predicting AKD.

Models	AUC	Precision	Recall	Accuracy	F1 score	Brier score	Matthewscorrelationcoefficient
**Training set**							
SVM	0.693 (0.682–0.704)	0.273 (0.147–0.398)	0.007 (0.004–0.011)	0.797 (0.796–0.798)	0.014 (0.007–0.022)	0.159 (0.159–0.160)	0.016 (0.000–0.036)
KNN	0.576 (0.568–0.585)	0.316 (0.293–0.339)	0.160 (0.149–0.172)	0.760 (0.755–0.766)	0.212 (0.198–0.227)	0.194 (0.190–0.197)	0.096 (0.077–0.114)
NB	0.727 (0.716–0.739)	0.365 (0.351–0.379)	0.559 (0.523–0.595)	0.715 (0.703–0.727)	0.441 (0.423–0.459)	0.266 (0.255–0.276)	0.272 (0.249–0.295)
MLP	0.697 (0.681–0.713)	0.459 (0.425–0.492)	0.323 (0.255–0.391)	0.783 (0.769–0.797)	0.369 (0.329–0.409)	0.161 (0.155–0.167)	0.256 (0.229–0.282)
RF	0.681 (0.672–0.690)	0.435 (0.416–0.454)	0.197 (0.187–0.208)	0.786 (0.782–0.790)	0.271 (0.259–0.283)	0.153 (0.152–0.155)	0.184 (0.170–0.198)
GBM	0.721 (0.711–0.730)	–	–	0.798 (0.798–0.798)	–	0.153 (0.153–0.154)	–
LR	0.679 (0.671–0.688)	0.469 (0.400–0.539)	0.045 (0.038–0.053)	0.797 (0.794–0.800)	0.083 (0.070–0.095)	0.152 (0.150–0.153)	0.094 (0.070–0.117)
LGBM	0.781 (0.770–0.793)	0.407 (0.396–0.418)	0.649 (0.629–0.669)	0.738 (0.731–0.746)	0.500 (0.487–0.513)	0.173 (0.170–0.177)	0.352 (0.334–0.370)
**Test set**							
SVM	0.695	0.394	0.015	0.798	0.028	0.158	0.042
KNN	0.587	0.303	0.156	0.758	0.206	0.192	0.087
NB	0.744	0.371	0.586	0.717	0.454	0.260	0.289
MLP	0.753	0.456	0.445	0.782	0.450	0.144	0.315
RF	0.689	0.448	0.084	0.795	0.141	0.152	0.122
GBM	0.720	-	-	0.799	-	0.153	-
LR	0.687	0.414	0.033	0.796	0.061	0.151	0.068
LGBM	0.781	0.400	0.649	0.734	0.495	0.175	0.346

*AUC, area under curve of the receiver operating characteristic curve.

**Figure 2:**
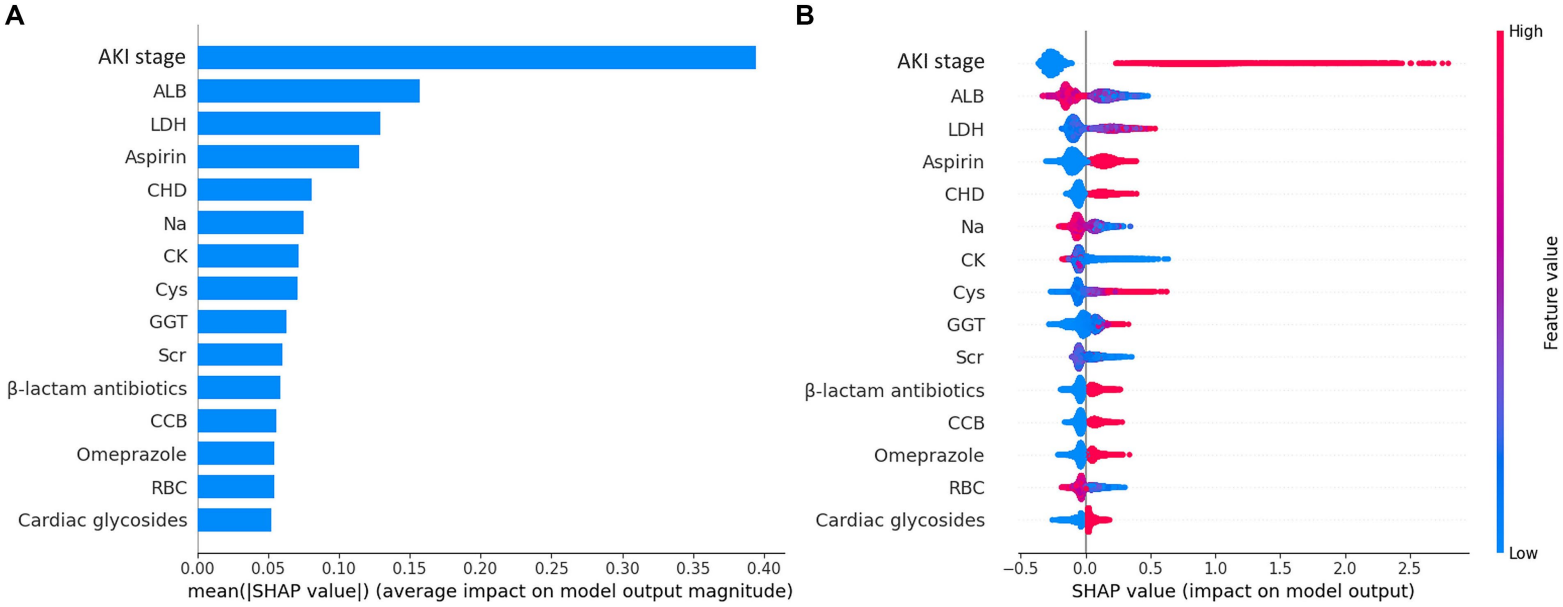
Importance matrix plot and SHAP summary plot of the final lite LGBM model. **(A)** The importance ranking of the first 15 features of the LGBM model. **(B)** The SHAP summary plot demonstrates the general importance of each feature in LGBM model. The color bar on the right indicates the relative value of a feature in each case. Red dots indicate high values and blue dots indicate low values. The violin graph lining up on the midline is the aggregation of dots representing each case in the train set. The distance between the upper and lower margin of the violin graph represents the amount of the cases that end up with the same SHAP values offered by this feature. SHAP force plots of 4 examples of patients. Categorical features including AKI stage, CHD, Omeprazole and β-lactam antibiotics were represented by 0 and 1, while “0” means “No” and “1” means “Yes.” *ALB, albumin; LDH, lactate dehydrogenase, CHD, coronary heart disease; CK, creatine kinase; Cys, cystatin C; GGT, gamma-glutamyl transferase; Scr, serum creatinine, CCB, calcium channel blocker; RBC, red blood cell count.

To identify the most significant features, we ranked the importance of LGBM features using the SHAP method in the training set. The evaluation metrics for LGBM models with different numbers of features were presented in Table 3. The model’s AUC increased to 0.760 when considering the top 15 features, leading to notable improvements in accuracy and precision. However, expanding the feature set to 20 did not yield a substantial uplift in AUC, and the other performance metrics exhibited a tendency toward stabilization. Given that, we selected the top 15 critical variables as the final lite prediction model for AKD (Figure 2A). Performance of the final lite LGBM model for AKD were presented in Supplementary Figure S3. We showed a DCA demonstrating the model’s substantial clinical utility. Furthermore, the confusion matrix, KS plot, and PR curve demonstrated the model exhibited satisfactory classification capabilities and maintained a favorable balance between precision and recall.

**Table 3:** Performance of LGBM model for predicting AKD.

Models	AUC	Precision	Recall	Accuracy	F1 score	Brier score	Matthews correlation coefficient
**Training set**							
Top 5 features	0.732 (0.718–0.747)	0.352 (0.339–0.365)	0.637 (0.612–0.663)	0.690 (0.679–0.702)	0.453 (0.438–0.469)	0.202 (0.198–0.205)	0.284 (0.262–0.307)
Top 10 features	0.754 (0.740–0.768)	0.369 (0.358–0.380)	0.662 (0.638–0.686)	0.703 (0.694–0.712)	0.474 (0.459–0.488)	0.192 (0.188–0.196)	0.314 (0.293–0.335)
Top 15 features	0.766 (0.752–0.780)	0.380 (0.368–0.391)	0.676 (0.652–0.701)	0.712 (0.703–0.720)	0.486 (0.472–0.500)	0.187 (0.183–0.191)	0.332 (0.311–0.353)
Top 20 features	0.773 (0.760–0.785)	0.379 (0.368–0.391)	0.685 (0.661–0.710)	0.710 (0.701–0.720)	0.488 (0.474–0.502)	0.188 (0.184–0.192)	0.335 (0.315–0.356)
All features	0.781 (0.770–0.793)	0.407 (0.396–0.418)	0.649 (0.629–0.669)	0.738 (0.731–0.746)	0.500 (0.487–0.513)	0.173 (0.170–0.177)	0.352 (0.334–0.370)
**Test set**							
Top 5 features	0.732	0.346	0.631	0.687	0.447	0.203	0.276
Top 10 features	0.746	0.351	0.658	0.688	0.458	0.197	0.292
Top 15 features	0.760	0.366	0.671	0.701	0.474	0.192	0.315
Top 20 features	0.766	0.372	0.673	0.706	0.479	0.190	0.323
All features	0.781	0.400	0.649	0.734	0.495	0.175	0.346

*AUC, area under curve of the receiver operating characteristic curve.

We employed the aforementioned methodology to derive features and construct models for both AKI and mortality prediction, with detailed results included in the supplementary files. The ROC curves utilizing all available features were illustrated in Figures 1B,C. The LGBM emerged as the optimal model for both AKI and mortality predictions (Supplementary Tables S2, S3), with 15 features identified as the ideal number for model performance (Supplementary Tables S4, S5). The refined model of AKI had an AUC of 0.767. In addition, it’s worth emphasizing that the final lite LGBM model of mortality showed impressive predictive capabilities, achieving an AUC of 0.927, and high recall and accuracy at 0.731 and 0.933, respectively. The ROC curves and DCA of the final lite LGBM model for AKI and mortality were presented in Supplementary Figures S4, S5.

### 3.3 Model interpretations

**Figure 3:**
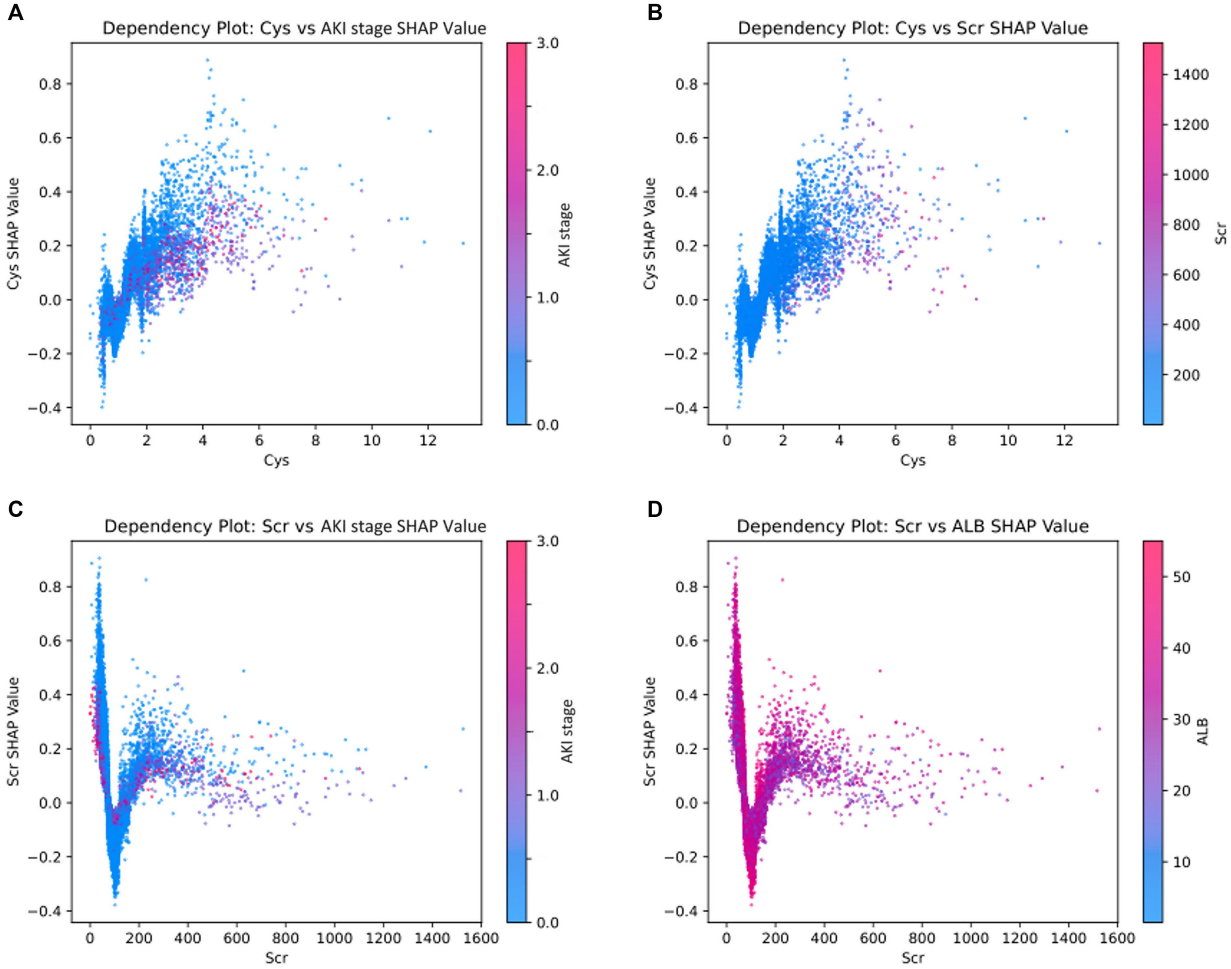
SHAP dependence plots demonstrate the distribution of SHAP output value of a single feature. The colors on the dependence plot correspond to another feature that could potentially interact with the feature being analyzed. **(A)** The relationship between Cys and AKI stage SHAP values, with the color bar indicating various levels of AKI stage. **(B)** The relationship between Cys and Scr SHAP values, where the color bar represents different levels of Scr. **(C)** The relationship between Scr and AKI stage SHAP values, with the color bar also denoting distinct AKI stage levels. **(D)** The relationship between Scr and ALB SHAP values, with the color bar reflecting varying ALB levels. *ALB, albumin; Cys, cystatin C; Scr, serum creatinine.

**Figure 4:**
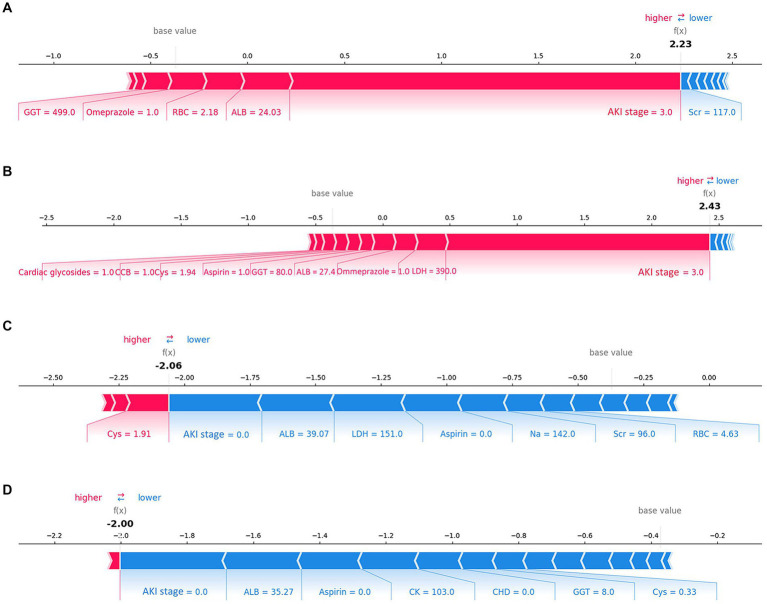
Force plots of the final lite LGBM model. **(A,B)** Show the examples of patients predicted to have AKD. **(C,D)** Show the examples of patients predicted to be non-AKD. The features shown in red represent a higher risk of AKD, while the features shown in blue represent a lower risk. The plots help physicians identify the main features in the model that have high decision power at the individual level. Categorical features including AKI stage, CHD, Omeprazole and *β*-lactam antibiotics were represented by 0 and 1, while “0” means “No” and “1” means “Yes.” *ALB, albumin; LDH, lactate dehydrogenase, CHD, coronary heart disease; CK, creatine kinase; Cys, cystatin C; GGT, gamma-glutamyl transferase; Scr, serum creatinine, CCB, calcium channel blocker; RBC, red blood cell count.

The SHAP summary plot (Figure 2B) displayed the contributions of the feature to the model. The analysis revealed that the primary factors influencing the model’s predictions were AKI stage, albumin (ALB), lactate dehydrogenase (LDH), the use of aspirin, and coronary heart disease (CHD). SHAP dependence plots (Figure 3) facilitated understanding how a single feature affected the output of the prediction model and showed the relationship between two features at the same time. For instance, as the value of Cys increased, so did the SHAP value and AKI stage, which implied a rising risk of developing AKD and a positive correlation between Cys and AKI stage (Figure 3A). The SHAP interaction plot (Supplementary Figure S6) revealed the interactions between all features. Furthermore, local explanation analyzed how features contributing to a particular prediction for an individual. The force plots (Figure 4) mainly presented the major factors that contributed to the final model output in a certain individual. Furthermore, the SHAP decision plots for other four patients (Supplementary Figure S7) provided a clear visualization of the decision-making paths attributed to each feature.

The SHAP method was also used for the AKI and mortality models, and detailed results were in the supplementary files. For the AKI model, Scr was the top contributing factor, as expected (Supplementary Figure S8). In the mortality model, the ‘dynamic’ variable ranked second in terms of significance (Supplementary Figure S9). The increasing ‘dynamic’ grade correlated with rising SHAP values, suggesting a higher mortality risk, highlighting the significant impact of kidney injury trajectory on older patients’ survival rates.

### 3.4 Online prediction website

Based on the lite prediction models, we developed an online risk website to streamline external validation and assess AKD and mortality risk in older patients. https://xuly94-elderly-hospitalized-patients-app-app-dxfrws.streamlit.app/, which can promptly generate the estimated risk for AKD and mortality offering immediate support for clinical decision-making.

### 3.5 Sensitivity analysis

The LGBM model demonstrated robust predictive accuracy for AKD stages 2–3, achieving an AUC of 0.843 in the test set (Supplementary Figure S10A). This indicated the model’s enhanced capability in predicting more severe cases of AKD, which was crucial to improve patient outcomes. When tested across various age groups, the performance of the model also remained stable (Supplementary Figures S10B–D). Specifically, the model yielded its highest performance in the 65–74 age subgroup, with an AUC of 0.755.

## 4 Discussion

In this retrospective cohort study, we developed and validated ML algorithms to forecast AKD, AKI, and mortality among older patients. The LGBM algorithm exhibited the strongest discrimination capability across all three outcomes. Additionally, SHAP was used for individualized patient interpretations, and an online AKD and mortality risk calculator for older patients was created, aiding early prediction and intervention. To the best of our knowledge, our study is the first to establish ML models for AKD, AKI and mortality in older patients that are valuable for risk assessment and clinical decision-making.

Several investigations have been conducted to explore the epidemiology of AKD before. James et al. reported that among more than one million Canadian residents, AKD without AKI was common — the incidence per 100 of the population tested was 3.8 in individuals without preexisting CKD and 0.6 in individuals with pre-existing CKD (22). Su et al. reported the incidence rate of community-acquired AKD was 4.60%, while it was 28.2% for hospital-acquired AKD (23). In our own study cohort, we observed that 4,434 patients, accounting for 20.15% of the total, satisfied the criteria for AKD.

In recent years, ML methods have been widely employed in predicting AKI (24–27). However, there is comparatively limited research on predicting AKD, particularly in older patients. A nomogram was developed and validated to predict the transition from AKI to AKD in patients undergoing partial nephrectomy for renal masses, demonstrating good discrimination with a concordance index of 0.891 (95% CI: 0.830, 0.953) (28). Chen et al. demonstrated that predictive models of acute decompensated heart failure (ADHF) patients had C-statistics of 0.726 (95% CI: 0.712–0.740) for AKD (29). Li et al. found that SVM showed better discrimination in older patients admitted to the intensive care unit (ICU) with AUC of 0.810 and 0.776 in the training and external validation cohorts, respectively (10). Unlike their study, which focused on older patients with AKD in the ICU, our research encompassed a broader spectrum, targeting the entire older patient population within hospital settings. What’s more, we have crafted models for predicting not only AKD but also AKI and mortality among older patients.

International consensus emphasizes the importance of early detection and prevention of AKD to mitigate its impact on patients and healthcare systems (9). Although, in theory, all older patients would benefit from comprehensive preventive measures against AKD, technical limitations often hinder early intervention. To address this issue, the ML algorithm simplifies early prediction. Furthermore, an online prediction website utilizing LGBM models can quickly identify high-risk older patients. This enables early detection and preventive interventions to enhance the prognosis for older individuals. The SHAP summary plot and force plots in Figure 2 enhanced understanding of the model’s decision-making process and can further assist physicians in implementing targeted preventive interventions for AKD.

In our study, the importance of variables showed that AKI stage, ALB, LDH, the use of aspirin and CHD were the most important factors that contributed to the predicted occurrence of AKD among older patients. Numerous studies have shown that AKI is intricately linked to the development of AKD (23, 29–31). Although current studies predominantly focus on AKI, they also suggest that these factors are risk for renal function impairment, consistent with our findings. Specifically, low serum albumin levels and elevated LDH levels are both associated with AKI AND poor outcomes (32–40). Aspirin, a common NSAID, and CHD have also been identified as independent risk factors for AKI, particularly among older people (34, 41–44).

This study has several key clinical implications. Firstly, it represents the initial effort to compare the baseline characteristics and hospital mortality across three distinct renal function trajectories post-injury. Secondly, we have successfully formulated succinct yet highly discriminative LGBM models for AKD, AKI, and mortality. Thirdly, the application of the SHAP method mitigated the opacity of ML models by globally and locally identifying and elucidating the most influential features for all three outcomes. In addition, we selected an optimal number of features for our final model to ensure a balance between complexity and clinical applicability, emphasizing its practicality with features that are readily obtainable in standard clinical settings. Furthermore, our models have been designed for direct clinical use, exemplified by a web-based risk calculator that assesses the risk of AKD and mortality in older patients, thus providing physicians with a valuable tool to enhance decision-making.

Our study faced several limitations. Firstly, it had a single-center design and a lack of ethnic diversity, which may affect the generalizability of our findings. Additionally, the identification of AKD and AKI could benefit from incorporating more early diagnostic markers, such as cystatin C, to improve predictive accuracy. Besides, the retrospective nature of our data collection introduces potential recall and selection biases. To address these issues, future research should aim for nationwide, multi-center prospective trials to enhance the validation and reliability of our predictive models, ensuring their applicability across diverse populations, including testing and verifying the model among people of other ethnicities. Last but not least, this article aims to predict kidney injury in older adult patients without specifically distinguishing the etiology. Due to the complex conditions of older adult patients, including numerous underlying diseases, susceptibility to infections, use of nephrotoxic drugs, and other common causes of kidney injury, it is often the result of multiple factors combined (8). Therefore, we have established a universal, comprehensive, and representative risk prediction model. However, its effectiveness in predicting kidney injury caused by different specific factors may not be optimal. Consequently, in future research, we plan to conduct separate studies on kidney injury caused by specific factors, such as sepsis.

This study highlights the increased susceptibility of older patients to AKD. We presented LGBM models to forecast AKD, AKI, and mortality at the time of admission. Furthermore, the web tool we developed to identify high-risk AKD and mortality cases in older patients can aid in clinical decision-making. Moving forward, we will conduct nationwide, multi-center trials with diverse participation, validating our predictive models across various ethnic groups.

## Data availability statement

The data underlying this article will be shared upon reasonable request to the corresponding author.

## Ethics statement

The study was approved by the Institutional Review Board (IRB; QYFY WZLL 28250) of the Affiliated Hospital of Qingdao University. The studies were conducted in accordance with the local legislation and institutional requirements. Written informed consent for participation was not required from the participants or the participants’ legal guardians/next of kin in accordance with the national legislation and institutional requirements.

## Author contributions

XW: Conceptualization, Data curation, Formal analysis, Investigation, Methodology, Validation, Writing – original draft, Writing – review & editing. LX: Writing – review & editing, Formal analysis, Methodology, Project administration, Software. CG: Formal analysis, Writing – review & editing, Data curation, Investigation. DX: Data curation, Formal analysis, Writing – review & editing. LC: Data curation, Writing – review & editing. YW: Data curation, Writing – review & editing. XM: Data curation, Writing – review & editing. CL: Data curation, Investigation, Project administration, Supervision, Validation, Writing – review & editing. YX: Funding acquisition, Project administration, Resources, Supervision, Validation, Visualization, Writing – review & editing.
