# Supplementary material for: Machine learning-based risk prediction of acute kidney disease and hospital mortality in older patients
Source: Front Med (Lausanne). 2024 Aug 15;11:1407354. doi: 10.3389/fmed.2024.1407354 (PMC11357947; doi:10.3389/fmed.2024.1407354)
Supplement: Supplementary file 1 [file Data_Sheet_1.docx]

Supplementary Material

Table of Contents

Supplementary Figure S1: Flow diagram of patient selection.

Supplementary Figure S2: Correlation matrix heatmap of eight ML models.

Supplementary Figure S3: Performance of the final lite LGBM model for AKD.

Supplementary Figure S4: Performance of the final lite LGBM model for AKI.

Supplementary Figure S5: Performance of the final lite LGBM model for mortality.

Supplementary Figure S6: SHAP interaction plot.

Supplementary Figure S7: SHAP decision plots.

Supplementary Figure S8: Feature importance and SHAP summary plot of the LGBM model for AKI.

Supplementary Figure S9: Feature importance and SHAP summary plot of the LGBM model for mortality.

Supplementary Figure S10: ROC of the final lite LGBM model for different groups of AKD.

Supplementary Table S1: Baseline characteristics of current cohort.

Supplementary Table S2: Performance of eight ML models for predicting AKI.

Supplementary Table S3: Performance of eight ML models for predicting mortality.

Supplementary Table S4: Performance of LGBM model for predicting AKI.

Supplementary Table S5: Performance of LGBM model for predicting mortality.

1. **Supplementary Figures and Tables**
   1. **Supplementary Figures**

**
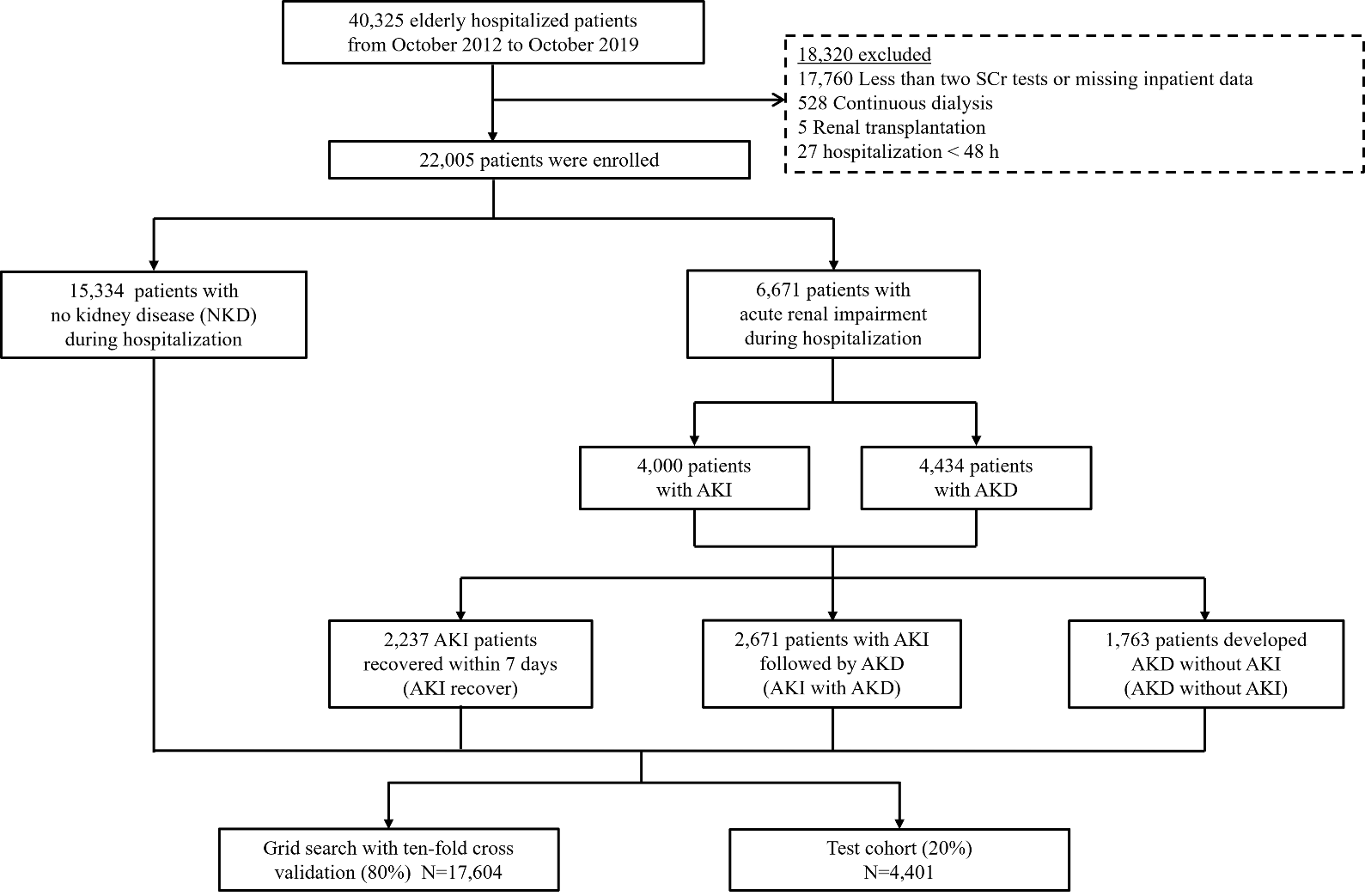
Supplementary Figure S1:** Flow diagram of patient selection.

**
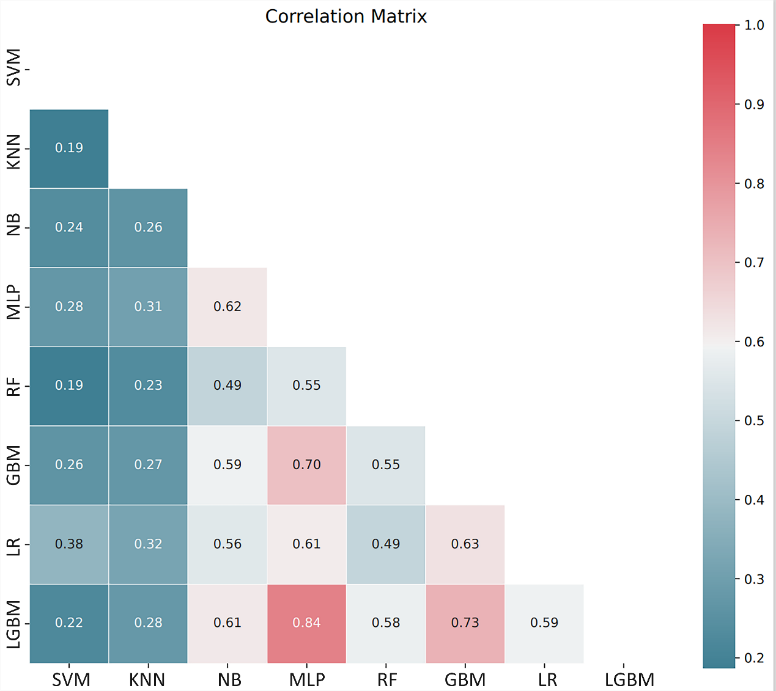
**

**Supplementary Figure S2:** Correlation matrix heatmap of eight ML models. The depth of the color represents the strength of the correlation, with darker colors (trending green) indicating lower correlation and lighter colors (trending red) indicating higher correlation.

**
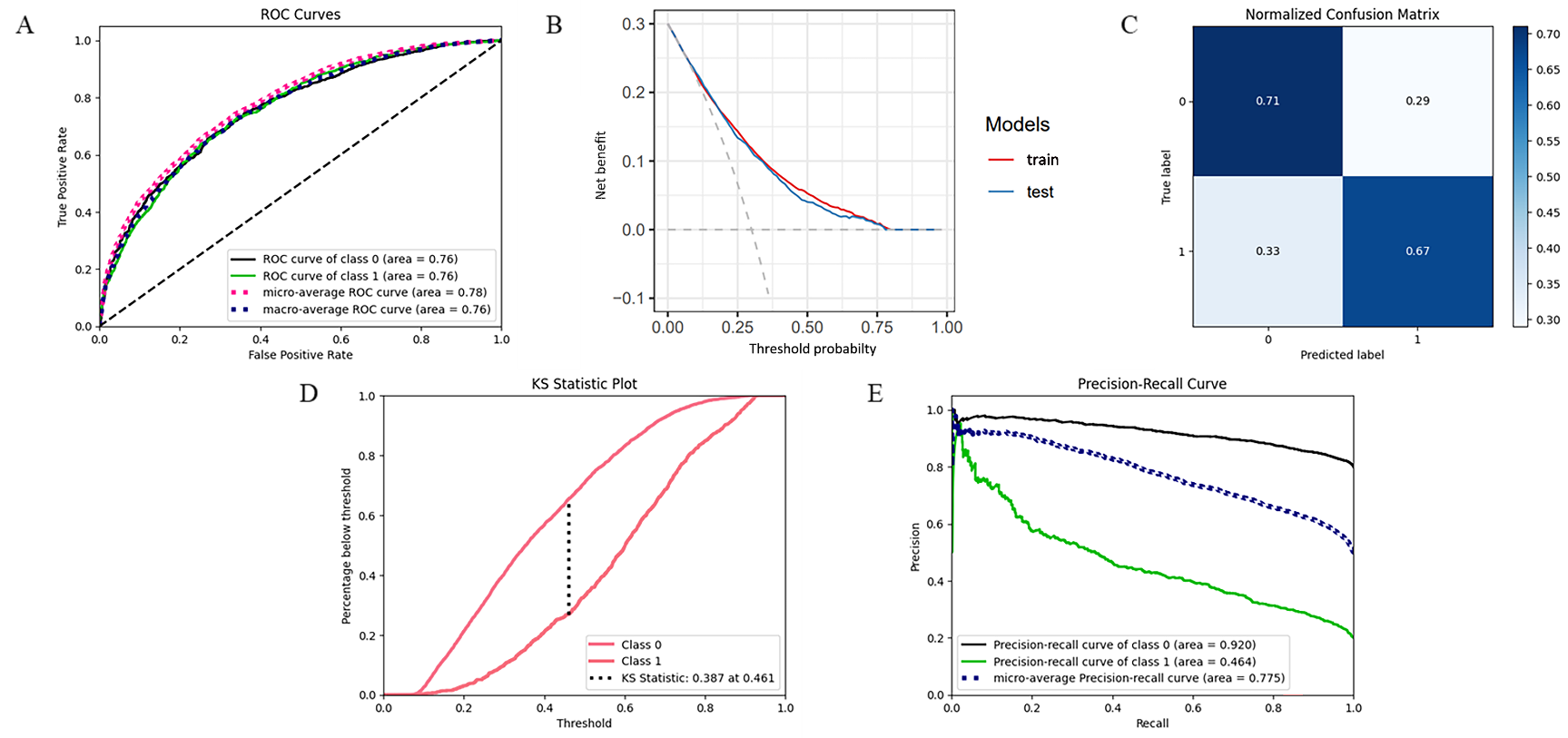
Supplementary Figure S3:** Performance of the final lite LGBM model for AKD. “Class 0” means “No AKD occurrence” and “Class 1” means “AKD occurrence”. **(A)** ROC curves of the final lite LGBM model in the test set. **(B)** DCA of the final lite LGBM model in the test set. **(C)** Confusion matrix of the final lite LGBM model in the test set. **(D)** KS plot of the final lite LGBM model in the test set. **(E)** PR curves of the final lite LGBM model in the test set.


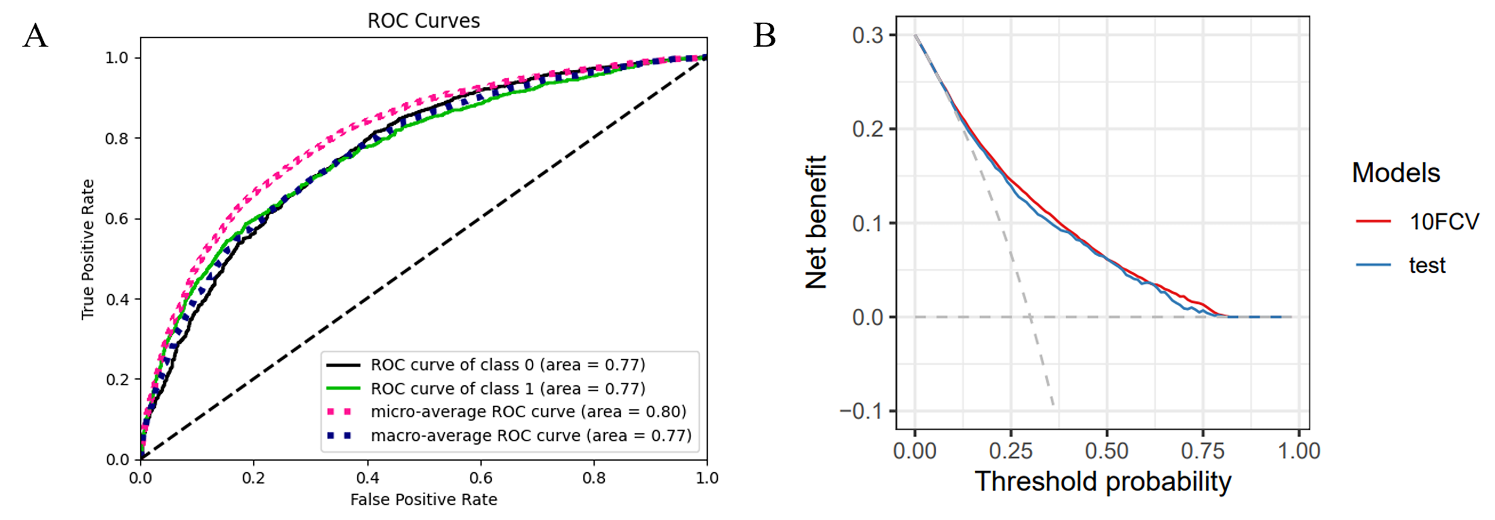
**Supplementary Figure S4:** Performance of the final lite LGBM model for AKI. **(A)** ROC curves of the final lite LGBM model for AKI. **(B)** DCA of the final lite LGBM model for AKI.


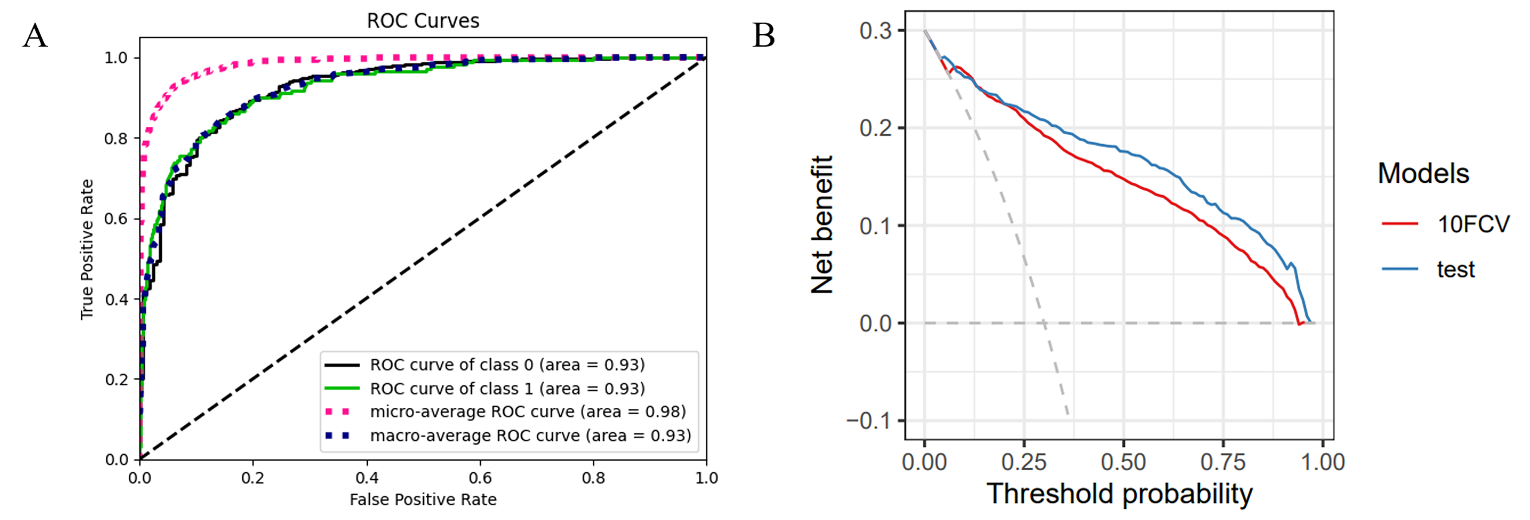
**Supplementary Figure S5:** Performance of the final lite LGBM model for mortality. **(A)** ROC curves of the final lite LGBM model for mortality. **(B)** DCA of the final lite LGBM model for mortality.

**
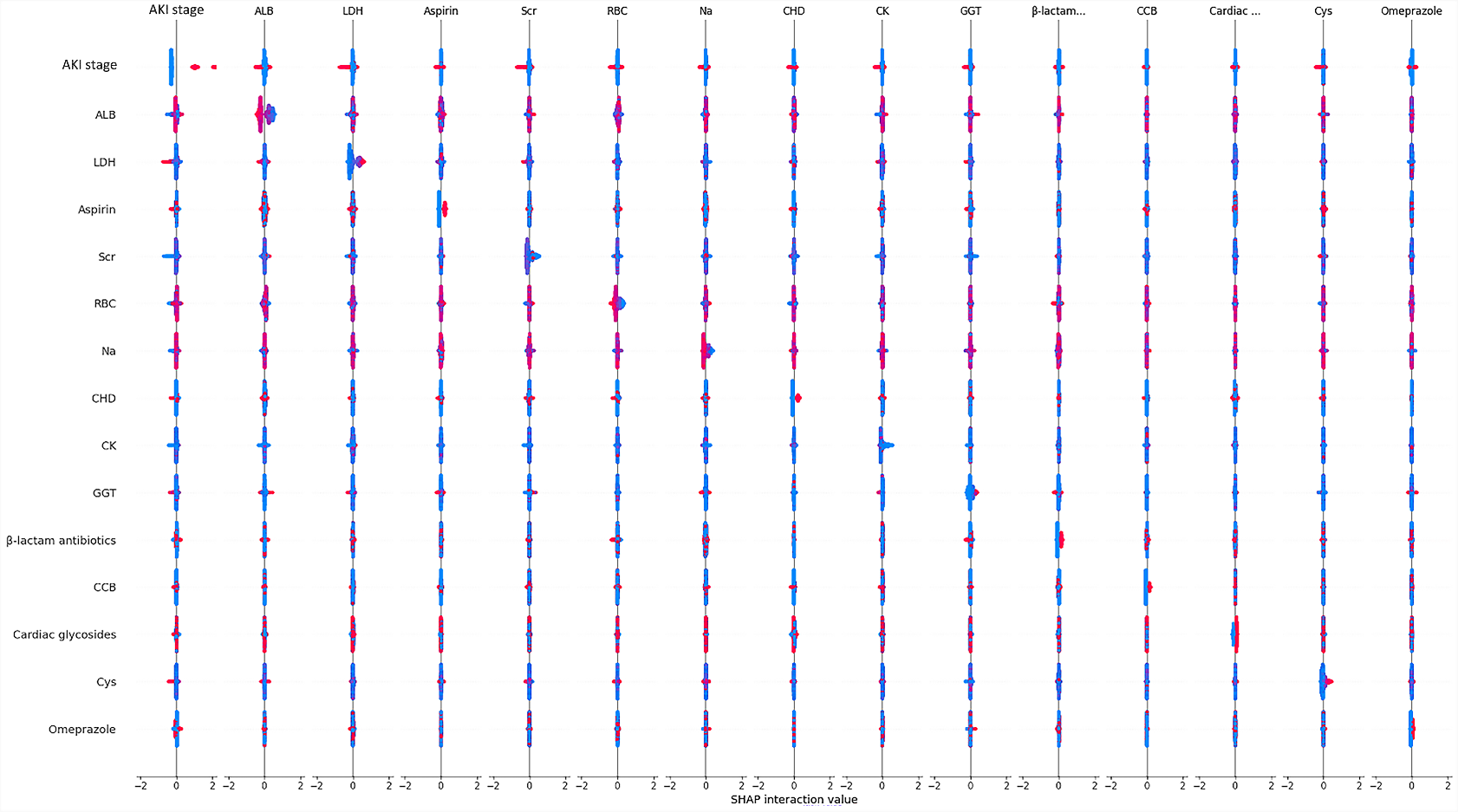
Supplementary Figure S6:** SHAP interaction plot. It illustrates the relative contributions of each feature to the output of the model and visualizes the pairwise interactions between features in the LGBM model. *ALB, albumin; LDH, lactate dehydrogenase, CHD, coronary heart disease; CK, creatine kinase; Cys, cystatin C; GGT, gamma-glutamyl transferase; Scr, serum creatinine, CCB, calcium channel blocker; RBC, red blood cell count.

**
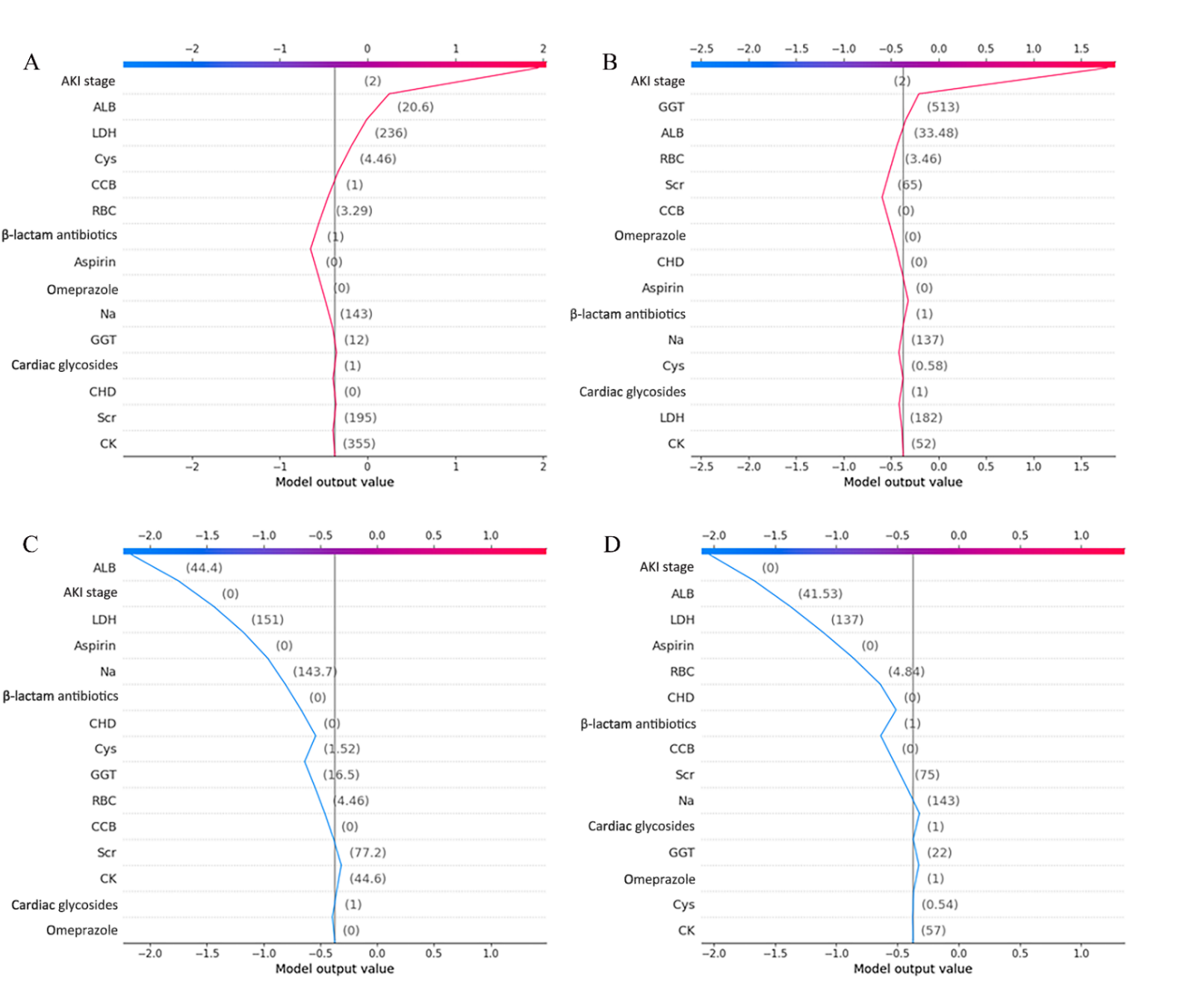
Supplementary Figure S7:** SHAP decision plots. The plots depict the decision path for predicting AKD and can better visualize the impact of each feature on the occurrence of AKD at the individual level. **(A)** and **(B)** show the examples of patients predicted to be non-AKD. **(C)** and **(D)** show the examples of patients predicted to have AKD. Categorical features including AKI stage, CHD, Omeprazole and β-lactam antibiotics were represented by 0 and 1, while “0” means “No” and “1” means “Yes”. * ALB, albumin; LDH, lactate dehydrogenase, CHD, coronary heart disease; CK, creatine kinase; Cys, cystatin C; GGT, gamma-glutamyl transferase; RBC, red blood cell count; CCB, calcium channel blocker.


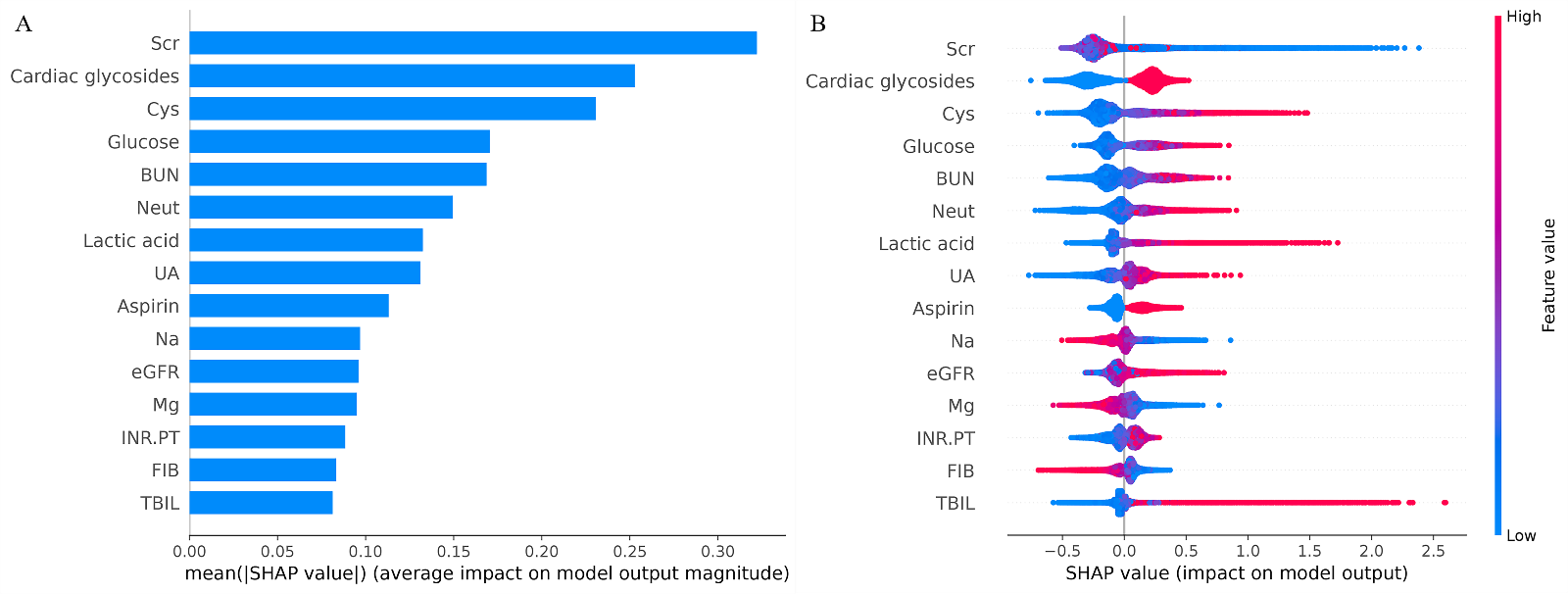
**Supplementary Figure S8:** Feature importance and SHAP summary plot of the LGBM model for AKI. **(A)** Feature importance ranking of the first 15 features. (**B)** SHAP summary plot of the first 15 features of the LGBM model. * Scr: serum creatinine; Cys, cystatin C; BUN, blood urea nitrogen; NEUT, neutrophil; UA, uric acid; eGFR, estimated glomerular filtration rate; INR.PT, international normalized ratio for prothrombin time; FIB, fibrinogen; TBIL, total bilirubin.


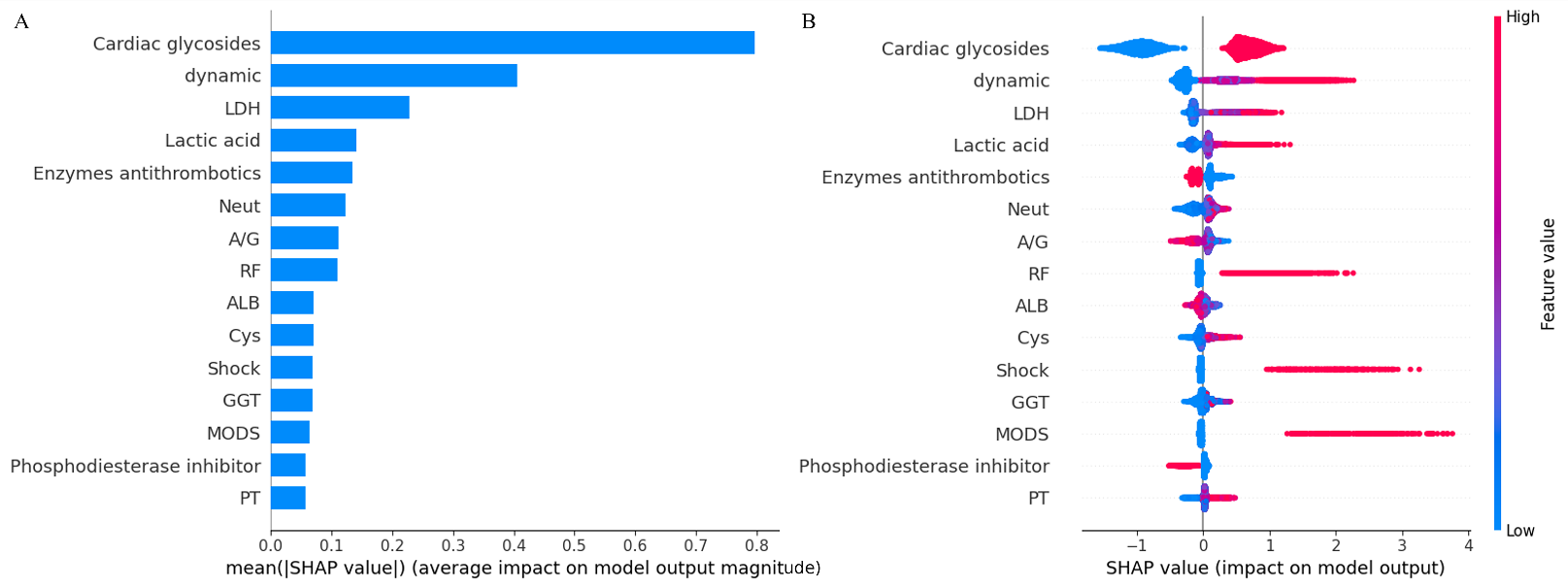
**Supplementary Figure S9:** Feature importance and SHAP summary plot of the LGBM model for mortality. **(A)** Feature importance ranking of the first 15 features. **(B)** SHAP summary plot of the first 15 features of the LGBM model. * LDH: lactate dehydrogenase; Neut: neutrophil count; A/G: albumin/globulin ratio; RF: respiratory failure; ALB: albumin; Cys, cystatin C; GGT: gamma glutamyl transferase; MODS: multiple organ dysfunction syndrome; PT: prothrombin time.


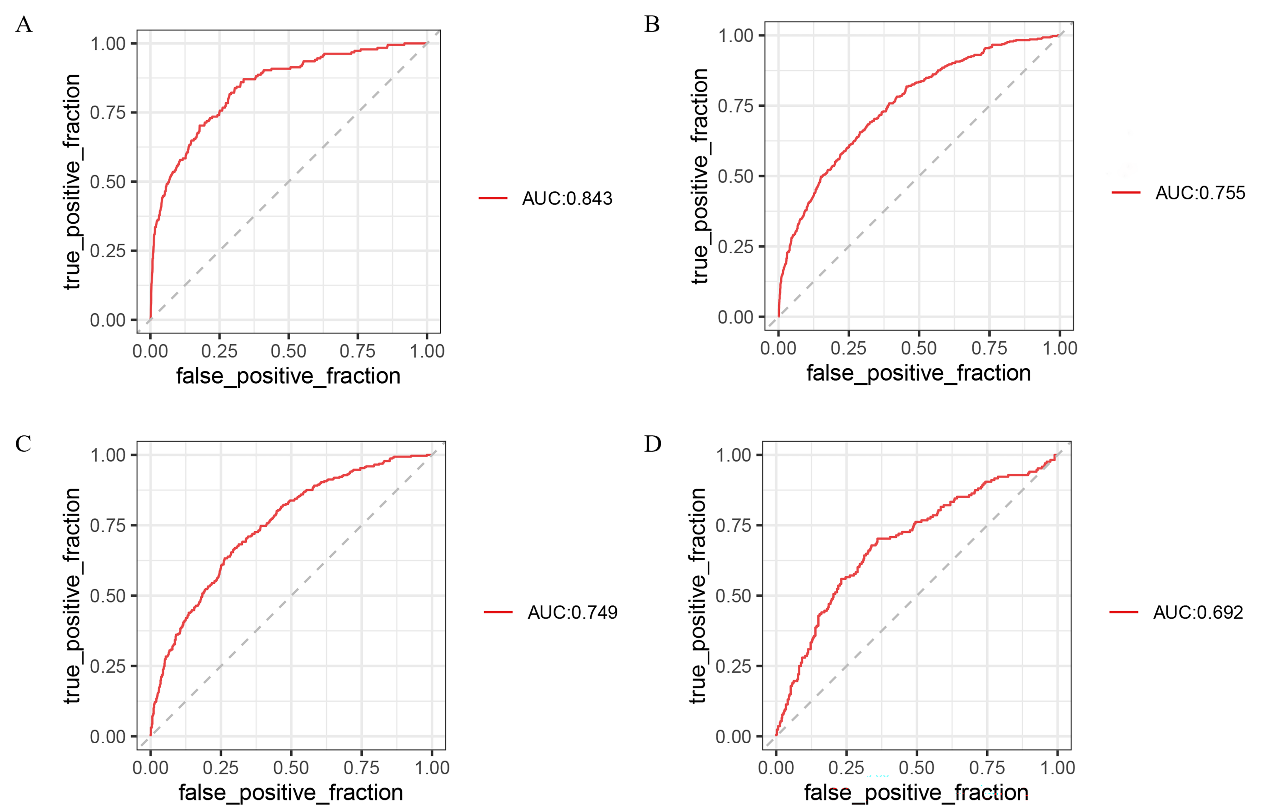
**Supplementary Figure S10**: ROC of the final lite LGBM model for different groups of AKD. **(A)** ROC curves of the final lite LGBM model for AKD 2-3 stage with AUC of 0.843 in the test set. **(B)** ROC curves of the final lite LGBM model for the age subgroup of 65-74 years old with AUC of 0.755 in the test set. **(C)** ROC curves of the final lite LGBM model for the age subgroup of 75-84 years old with AUC of 0.749 in the test set. **(D)** ROC curves of the final lite LGBM model for the age subgroup of ≥85years old with AUC of 0.692 in the test set.

- 1. **Supplementary Tables**

| **Supplementary Table S1.** Baseline characteristics of current cohort. | | | | | | | |
| --- | --- | --- | --- | --- | --- | --- | --- |
| Variables | NKD  (n = 15 334) | Acute/subacute renal impairment | | | | | |
|  |  | AKI recovery  (n = 2237) | AKD without AKI (n = 2671) | AKD with AKI  (n = 1763) | Total  (N = 6671) | \|t/Z/χ2\| | P-value |
| **Demographics** |  |  | | | | | |
| Age (years) | 73.00 (12.00) | 75.00 (13.00) | 76.00 (14.00) | 75.00 (12.00) | 75.00 (13.00) | 11.731 | <0.001 |
| Male, n (%) | 9474 (61.80) | 1308 (58.50) | 1473 (55.10) | 1023 (58.00) | 3804 (57.00) | 44.038 | <0.001 |
| BMI (kg/m^2^) | 23.71 (5.06) | 24.00 (45.30) | 23.44 (5.19) | 23.66 (5.34) | 23.66 (5.22) | 1.884 | 0.060 |
| SBP (mmHg) | 132.00 (28.00) | 133.00 (30.00) | 131.00 (28.00) | 130.00 (29.00) | 131.00 (29.00) | 2.536 | 0.011 |
| DBP (mmHg) | 78.00 (14.00) | 78.00 (17.00) | 75.00 (15.00) | 76.00 (18.00) | 76.00 (17.00) | 4.808 | <0.001 |
| Smoke, n (%) | 5738 (37.40) | 741 (33.10) | 856 (32.00) | 574 (32.60) | 2171 (32.50) | 48.009 | <0.001 |
| Drink, n (%) | 4227 (27.60) | 532 (23.80) | 635 (23.80) | 440 (25.00) | 1607 (24.10) | 28.843 | <0.001 |
| Transfusion, n (%) | 1920 (12.50) | 339 (15.20) | 574 (21.50) | 382 (21.70) | 1295 (19.40) | 176.949 | <0.001 |
| Medicine, n (%) | 12846 (83.80) | 1923 (86.00) | 2405 (90.00) | 1558 (88.40) | 5886 (88.20) | 72.967 | <0.001 |
| Allergy, n (%) | 2306 (15.00) | 274 (12.20) | 442 (16.50) | 257 (14.60) | 973 (14.60) | 0.753 | 0.386 |
| **Laboratory data** |  | | | | | | |
| Scr (umol/L) | 83.00 (27.92) | 82.00 (59.00) | 82.00 (41.00) | 83.00 (69.80) | 82.00 (52.73) | 1.835 | 0.066 |
| eGFR (ml/min/1.73^2^) | 70.96 (20.42) | 68.36 (32.93) | 68.75 (26.45) | 67.77 (33.69) | 68.36 (30.72) | 6.322 | <0.001 |
| BUN (mmol/L) | 5.78 (2.89) | 6.70 (5.59) | 6.14 (4.49) | 6.92 (6.42) | 6.52 (5.22) | 19.940 | <0.001 |
| Cys (mg/L) | 1.00 (0.46) | 1.08 (0.87) | 1.09 (0.69) | 1.23 (1.13) | 1.12 (0.82) | 21.540 | <0.001 |
| Glucose (mmol/L) | 5.53 (2.15) | 6.50 (3.90) | 5.92 (2.79) | 6.50 (3.78) | 6.24 (3.42) | 22.283 | <0.001 |
| Na (mmol/L) | 140.18 (4.69) | 138.65 (6.45) | 138.75 (5.58) | 138.28 (6.47) | 138.59 (6.12) | 18.876 | <0.001 |
| P (mmol/L) | 1.04 (0.25) | 1.07 (0.36) | 1.02 (0.35) | 1.05 (0.40) | 1.04 (0.37) | 0.014 | 0.989 |
| Lipoprotein (a) (mg/L) | 201.40 (293.60) | 188.00 (296.00) | 208.00 (311.50) | 181.00 (296.00) | 193.00 (302.30) | 2.926 | 0.003 |
| Ca (mmol/L) | 2.16 (0.20) | 2.09 (0.22) | 2.09 (0.21) | 2.07 (0.23) | 2.08 (0.22) | 21.917 | <0.001 |
| K (mmol/L) | 4.18 (0.51) | 4.22 (0.69) | 4.09 (0.62) | 4.20 (0.74) | 4.17 (0.68) | 1.381 | 0.167 |
| Mg (mmol/L) | 0.91 (0.11) | 0.90 (0.14) | 0.89 (0.13) | 0.90 (0.14) | 0.90 (0.14) | 6.530 | <0.001 |
| CK (U/L) | 66.00 (64.48) | 73.00 (93.05) | 55.00 (70.00) | 71.00 (108.00) | 65.00 (88.40) | 0.392 | 0.695 |
| Hb (g/L) | 122.02 (23.48) | 118.94 (27.07) | 112.81 (25.21) | 112.75 (26.46) | 114.85 (26.33) | 19.161 | <0.001 |
| PLT (10^9^/L) | 225.98 (95.49) | 207.07 (89.12) | 217.94 (102.33) | 204.51 (95.89) | 210.74 (96.54) | 10.793 | <0.001 |
| RBC (10^12^/L) | 4.08 (0.71) | 3.96 (0.87) | 3.80 (0.80) | 3.77 (0.86) | 3.85 (0.84) | 19.962 | <0.001 |
| WBC (10^9^/L) | 6.42 (3.17) | 7.42 (5.09) | 7.06 (4.18) | 7.50 (5.10) | 7.33 (4.73) | 21.405 | <0.001 |
| Neut (10^9^/L) | 3.94 (2.83) | 5.17 (5.32) | 4.84 (4.02) | 5.38 (5.07) | 5.07 (4.77) | 28.185 | <0.001 |
| ALT (U/L) | 17.00 (15.00) | 19.00 (23.00) | 18.00 (20.00) | 21.00 (32.00) | 19.00 (23.50) | 11.193 | <0.001 |
| GGT (U/L) | 20.00 (25.00) | 21.40 (36.65) | 25.00 (41.00) | 28.00 (63.70) | 25.00 (43.00) | 15.013 | <0.001 |
| TBIL (umol/L) | 13.40 (9.30) | 14.20 (12.68) | 13.90 (12.50) | 15.80 (17.07) | 14.50 (13.46) | 10.485 | <0.001 |
| AST (U/L) | 18.00 (11.00) | 20.00 (22.00) | 20.00 (17.00) | 23.00 (29.00) | 20.50 (21.00) | 18.597 | <0.001 |
| TC (mmol/L) | 4.67 (1.43) | 4.42 (1.51) | 4.42 (1.55) | 4.43 (1.91) | 4.42 (1.64) | 10.997 | <0.001 |
| HDL (mmol/L) | 1.23 (0.41) | 1.15 (0.48) | 1.14 (0.46) | 1.08 (0.55) | 1.13 (0.49) | 14.933 | <0.001 |
| LDH (U/L) | 163.00 (60.00) | 179.00 (93.00) | 181.00 (93.00) | 193.00 (105.00) | 183.60 (96.00) | 25.053 | <0.001 |
| UA (umol/L) | 291.82 (114.30) | 323.06 (161.04) | 293.09 (145.84) | 318.84 (175.47) | 309.55 (159.83) | 8.192 | <0.001 |
| LDL (mmol/L) | 2.61 (1.25) | 2.38 (1.30) | 2.35 (1.30) | 2.28 (1.35) | 2.34 (1.31) | 16.083 | <0.001 |
| A/G (mmol/L) | 1.26 (0.34) | 1.22 (0.43) | 1.15 (0.33) | 1.17 (0.33) | 1.18 (0.37) | 16.356 | <0.001 |
| TG (mmol/L) | 1.05 (0.71) | 1.07 (0.84) | 1.06 (0.71) | 1.10 (0.84) | 1.07 (0.78) | 2.748 | 0.006 |
| ALP (U/L) | 72.00 (34.14) | 70.00 (39.45) | 73.60 (42.00) | 74.70 (52.00) | 73.00 (43.00) | 3.063 | 0.002 |
| TP (g/L) | 63.84 (8.23) | 61.22 (8.93) | 61.24 (9.02) | 60.44 (9.44) | 63.84 (8.23) | 21.659 | <0.001 |
| ALB (g/L) | 35.09 (6.07) | 33.19 (6.53) | 32.07 (6.32) | 32.07 (6.54) | 32.44 (6.47) | 28.366 | <0.001 |
| PT (s) | 10.50 (2.20) | 11.30 (3.00) | 11.10 (2.50) | 11.40 (3.00) | 11.20 (2.80) | 26.097 | <0.001 |
| FIB (g/L) | 3.53 (1.09) | 3.56 (1.19) | 3.62 (1.20) | 3.59 (1.24) | 3.59 (1.21) | 3.544 | <0.001 |
| TT (s) | 14.50 (3.20) | 14.90 (3.60) | 15.00 (3.60) | 15.00 (3.60) | 15.00 (3.60) | 11.825 | <0.001 |
| HCT (%) | 36.81 (7.02) | 35.80 (8.28) | 34.35 (7.72) | 34.12 (8.23) | 34.78 (8.08) | 17.853 | <0.001 |
| Lactic acid (mmol/L) | 1.20 (0.70) | 1.40 (1.00) | 1.20 (0.80) | 1.40 (1.10) | 1.30 (0.90) | 13.383 | <0.001 |
| HbAlc (%) | 6.92 (1.66) | 7.06 (1.88) | 6.09 (1.69) | 6.94 (1.76) | 6.96 (1.78) | 1.902 | 0.057 |
| INR.PT | 0.96 (0.14) | 1.00 (0.18) | 1.00 (0.16) | 1.02 (0.18) | 1.01 (0.18) | 25.366 | <0.001 |
| C1q (mg/L) | 180.13 (46.03) | 175.88 (46.38) | 176.84 (46.70) | 173.64 (51.01) | 175.67 (47.78) | 6.524 | <0.001 |
| CRP (mg/L) | 6.44 (34.60) | 13.64 (59.65) | 14.94 (50.65) | 18.36 (60.46) | 15.34 (56.87) | 20.112 | <0.001 |
| **Comorbidities, n %** |  |  | | | | | |
| CKD | 630 (4.10) | 181 (8.10) | 210 (7.90) | 249 (14.10) | 640 (9.60) | 257.189 | <0.001 |
| NS | 169 (1.10) | 2 (0.10) | 38 (1.40) | 16 (1.90) | 56 (0.80) | 3.169 | 0.075 |
| Nephritis | 97 (0.60) | 6 (0.30) | 18 (0.70) | 12 (0.70) | 36 (0.50) | 0.668 | 0.414 |
| Renal calculi | 401 (2.60) | 67 (3.00) | 59 (2.20) | 63 (3.60) | 189 (2.80) | 0.847 | 0.357 |
| Hydronephrosis | 144 (0.90) | 39 (1.70) | 29 (1.10) | 23 (1.30) | 91 (1.40) | 7.948 | 0.005 |
| Cystic kidney disease | 939 (6.10) | 115 (5.10) | 163 (6.10) | 94 (5.30) | 372 (5.60) | 2.485 | 0.115 |
| Respiratory failure | 514 (3.40) | 128 (5.70) | 235 (8.80) | 172 (9.80) | 535 (8.00) | 223.101 | <0.001 |
| Bronchitis | 375 (2.40) | 32 (1.40) | 69 (2.60) | 27 (1.50) | 128 (1.90) | 5.776 | 0.016 |
| Asthma | 327 (2.10) | 30 (1.30) | 56 (2.10) | 33 (1.90) | 119 (1.80) | 2.846 | 0.092 |
| Pulmonary emphysema | 557 (3.60) | 41 (1.80) | 89 (3.30) | 46 (2.60) | 176 (2.60) | 14.268 | <0.001 |
| COPD | 682 (4.40) | 76 (3.40) | 179 (6.70) | 81 (4.60) | 336 (5.00) | 3.656 | 0.056 |
| Intracerebral hemorrhage | 349 (2.30) | 55 (2.50) | 124 (2.60) | 63 (3.60) | 242 (3.60) | 32.495 | <0.001 |
| Cerebral infarction | 1598 (10.40) | 202 (9.00) | 386 (14.50) | 192 (10.90) | 780 (11.70) | 7.792 | 0.005 |
| Hepatitis | 405 (2.60) | 28 (1.30) | 62 (2.30) | 38 (2.20) | 128 (1.90) | 10.265 | 0.001 |
| Fatty liver disease | 456 (3.00) | 38 (1.70) | 47 (1.80) | 34 (1.90) | 119 (1.80) | 25.866 | <0.001 |
| Cholelithiasis | 1375 (9.00) | 133 (5.90) | 234 (8.80) | 126 (7.10) | 493 (7.40) | 14.878 | <0.001 |
| Peptic ulcer | 847 (5.50) | 86 (3.80) | 131 (4.90) | 67 (3.80) | 284 (4.30) | 15.292 | <0.001 |
| Hepatic cirrhosis | 331 (2.20) | 22 (1.00) | 79 (3.00) | 50 (2.80) | 151 (2.30) | 0.239 | 0.625 |
| Diabetes | 3296 (21.50) | 469 (21.00) | 688 (25.80) | 448 (25.40) | 1605 (24.10) | 17.662 | <0.001 |
| Hyperuricemia | 286 (1.90) | 43 (1.90) | 74 (2.80) | 32 (1.80) | 149 (2.20) | 3.256 | 0.071 |
| Hypoproteinemia | 409 (2.70) | 53 (2.40) | 152 (5.70) | 84 (4.80) | 289 (4.30) | 41.954 | <0.001 |
| Atrial fibrillation | 221 (1.40) | 69 (3.10) | 134 (5.00) | 103 (5.80) | 306 (4.60) | 196.797 | <0.001 |
| Acute coronary syndrome | 1449 (9.40) | 339 (15.20) | 427 (16.00) | 303 (17.20) | 1069 (16.00) | 198.316 | <0.001 |
| Coronary heart disease | 4153 (27.10) | 662 (29.60) | 1052 (39.40) | 619 (35.10) | 2333 (35.00) | 139.166 | <0.001 |
| Shock | 87 (0.60) | 77 (3.40) | 48 (1.80) | 140 (7.90) | 265 (4.00) | 342.416 | <0.001 |
| Hypertension | 6611 (43.10) | 919 (41.10) | 1328 (49.70) | 807 (45.80) | 3054 (45.80) | 13.423 | <0.001 |
| MODS | 48 (0.30) | 71 (3.20) | 45 (1.70) | 160 (9.10) | 276 (4.10) | 468.644 | <0.001 |
| Septicemia | 65 (0.40) | 10 (0.40) | 23 (0.90) | 18 (1.00) | 51 (0.80) | 10.285 | 0.001 |
| **Medications, n %** |  | | | | | | |
| β-receptor blocker | 5539 (36.10) | 791 (35.40) | 962 (36.00) | 810 (45.90) | 2563 (38.40) | 10.550 | 0.001 |
| ACEI | 1242 (8.10) | 249 (11.10) | 390 (14.60) | 247 (14.00) | 886 (13.30) | 142.887 | <0.001 |
| ARB | 3059 (19.90) | 418 (18.70) | 661 (24.70) | 372 (21.10) | 1451 (21.80) | 9.261 | 0.002 |
| Statin | 3786 (24.70) | 592 (26.50) | 829 (31.00) | 504 (28.60) | 1925 (28.90) | 41.983 | <0.001 |
| CCB | 4901 (32.00) | 737 (32.90) | 1034 (38.70) | 732 (41.50) | 2503 (37.50) | 64.344 | <0.001 |
| β-lactam antibiotics | 5629 (36.70) | 920 (41.10) | 1276 (47.80) | 978 (55.50) | 3174 (47.60) | 228.844 | <0.001 |
| Macrolides antibiotics | 516(3.4) | 35(1.6) | 60(2.2) | 35(2.0) | 130(1.9) | 32.726 | <0.001 |
| Aminoglycosides antibiotics | 586(3.8) | 76(3.4) | 124(4.6) | 91(5.2) | 291(4.4) | 3.550 | 0.06 |
| Quinolones antibiotics | 3935 (25.70) | 515 (23.00) | 878 (32.90) | 525 (29.80) | 1918 (28.80) | 22.725 | <0.001 |
| Cardiac glycosides | 8209 (53.50) | 1371 (61.30) | 1502 (56.20) | 1270 (72.00) | 4143 (62.10) | 138.653 | <0.001 |
| Phosphodiesterase inhibitor | 1450 (9.50) | 197 (8.80) | 260 (9.70) | 229 (13.00) | 686 (10.30) | 3.629 | 0.057 |
| Biguanides | 975 (6.40) | 115 (5.10) | 173 (6.50) | 112 (6.40) | 400 (6.00) | 1.042 | 0.307 |
| Sulfonylureas | 501 (3.30) | 50 (2.20) | 93 (3.50) | 57 (3.20) | 200 (3.00) | 1.092 | 0.296 |
| Glucosidase Inhibitors | 1216 (7.90) | 131 (5.90) | 253 (9.50) | 128 (7.30) | 512 (7.70) | 0.418 | 0.518 |
| HMG-CoA Reductase Inhibitors | 3786 (24.70) | 592 (26.50) | 829 (31.00) | 504 (28.60) | 1925 (28.90) | 41.983 | <0.001 |
| Vitamin K antagonist | 608 (4.00) | 149 (6.70) | 196 (7.30) | 153 (8.70) | 498 (7.50) | 119.302 | <0.001 |
| Platelet Aggregation Inhibitors | 3910 (25.50) | 659 (29.50) | 935 (35.00) | 597 (33.90) | 2191 (32.80) | 125.146 | <0.001 |
| Enzymes anti-thrombotic | 7419 (48.40) | 811 (36.30) | 875 (32.80) | 766 (43.40) | 2452 (36.80) | 254.042 | <0.001 |
| Factor Xa inhibitor | 435 (2.80) | 52 (2.30) | 38 (1.40) | 20 (1.10) | 110 (1.60) | 27.158 | <0.001 |
| Dipyridamole | 826(5.4) | 112(5.0) | 199(7.5) | 93(5.3) | 404(6.1) | 3.947 | 0.047 |
| Aspirin | 5461(35.6) | 929(41.5) | 1201(45.0) | 933(52.9) | 3063(45.9) | 202.865 | <0.001 |
| Pantoprazole | 6094 (39.70) | 1063 (47.50) | 1084 (40.60) | 826 (46.90) | 2973 (44.60) | 44.658 | <0.001 |
| Omeprazole | 6396 (41.70) | 796 (35.60) | 1254 (46.90) | 868 (49.20) | 2918 (43.70) | 7.850 | 0.005 |
| Esomeprazole | 2172 (14.20) | 295 (13.20) | 478 (14.90) | 379 (21.50) | 1152 (17.30) | 34.930 | <0.001 |
| Rabeprazole | 1339 (8.70) | 165 (7.40) | 252 (9.40) | 133 (7.50) | 550 (8.20) | 1.408 | 0.235 |
| Lansoprazole | 5345 (34.90) | 747 (33.40) | 1066 (39.90) | 665 (37.70) | 2478 (37.10) | 10.627 | <0.001 |
| **Outcomes** |  | | | | | | |
| Hospital mortality (n, %) | 223 (1.50) | 167 (7.50) | 140 (5.20) | 336 (19.10) | 643 (9.60) | 823.653 | <0.001 |
| LOS (days) | 17.00 (9.00) | 14.00 (11.00) | 20.00 (12.00) | 20.00 (16.00) | 18.00 (14.00) | 2.691 | 0.007 |

Values are presented as mean with standard deviation, or median with interquartile range unless stated otherwise. The P-value was calculated among NKD and three subtypes of acute/subacute renal impairment. * BMI: body mass index; SBP: systolic blood pressure; DBP: diastolic blood pressure; Scr: serum creatinine; eGFR: estimated glomerular filtration rate; BUN: blood urea nitrogen; Cys, cystatin C; CK: creatine kinase; Hb: hemoglobin; PLT: platelet; RBC: red blood cell; WBC: white blood cell; Neut: neutrophil count; ALT: alanine aminotransferase; GGT: gamma glutamyl transferase; TBIL: total bilirubin; AST: aspartate aminotransferase; TC: total cholesterol; HDL: high-density lipoprotein; LDH: lactate dehydrogenase; UA: uric acid; LDL: low-density lipoprotein; A/G: albumin/globulin ratio; TG: triglycerides; ALP: alkaline phosphatase; TP: total protein; ALB: albumin; PT: prothrombin time; FIB: fibrinogen; TT: thrombin time; HCT: hematocrit; Lactic acid: lactic acid; HbA1c: hemoglobin A1c; INR.PT: international normalized ratio for prothrombin time; C1q: complement component 1q; CRP: C-reactive protein; CKD: chronic kidney disease; NS: nephrotic syndrome; COPD: chronic obstructive pulmonary disease; MODS: multiple organ dysfunction syndrome; ACEI: angiotensin-converting enzyme inhibitors; ARB: angiotensin II receptor blockers; CCB: calcium channel blockers; LOS: length of stay.

| **Supplementary Table S2.** Performance of eight ML models for predicting AKI. | | | | | | | |
| --- | --- | --- | --- | --- | --- | --- | --- |
| Models | AUC | Precision | Recall | Accuracy | F1 score | Brier Score | Matthews  Correlation  Coefficient |
| **Training set** |  |  |  |  |  |  |  |
| SVM | 0.712 (0.702-0.723) | 0.670 (0.628-0.712) | 0.082 (0.070-0.093) | 0.825 (0.824-0.827) | 0.145 (0.128-0.162) | 0.140 (0.139-0.140) | 0.189 (0.175-0.203) |
| KNN | 0.588 (0.573-0.603) | 0.355 (0.327-0.384) | 0.159 (0.143-0.175) | 0.794 (0.789-0.800) | 0.219 (0.199-0.238) | 0.174 (0.170-0.178) | 0.133 (0.111-0.155) |
| NB | 0.725 (0.714-0.736) | 0.454 (0.436-0.473) | 0.352 (0.333-0.371) | 0.805 (0.800-0.811) | 0.396 (0.379-0.414) | 0.189 (0.184-0.194) | 0.286 (0.266-0.306) |
| MLP | 0.701 (0.686-0.717) | 0.460 (0.430-0.489) | 0.379 (0.328-0.430) | 0.804 (0.792-0.817) | 0.411 (0.378-0.443) | 0.154 (0.147-0.160) | 0.300 (0.270-0.330) |
| RF | 0.703 (0.696-0.711) | 0.496 (0.472-0.521) | 0.217 (0.203-0.232) | 0.818 (0.814-0.821) | 0.302 (0.284-0.320) | 0.137 (0.135-0.138) | 0.240 (0.220-0.260) |
| GBM | 0.703 (0.692-0.714) | 0.100 (0.000-0.326) | 0.000 (0.000-0.001) | 0.818 (0.818-0.818) | 0.001 (0.000-0.002) | 0.142 (0.142-0.143) | 0.004(0.000-0.016) |
| LR | 0.722 (0.715-0.730) | 0.593 (0.553-0.633) | 0.125 (0.109-0.140) | 0.825 (0.822-0.828) | 0.205 (0.183-0.227) | 0.132 (0.131-0.134) | 0.212 (0.189-0.235) |
| LGBM | 0.806 (0.797-0.816) | 0.485 (0.470-0.499) | 0.559 (0.544-0.575) | 0.812 (0.806-0.817) | 0.519 (0.506-0.533) | 0.135 (0.132-0.138) | 0.404 (0.387-0.421) |
| **Test set** |  |  |  |  |  |  |  |
| SVM | 0.704 | 0.631 | 0.066 | 0.823 | 0.120 | 0.142 | 0.163 |
| KNN | 0.597 | 0.356 | 0.148 | 0.797 | 0.209 | 0.170 | 0.129 |
| NB | 0.714 | 0.436 | 0.318 | 0.802 | 0.368 | 0.194 | 0.258 |
| MLP | 0.743 | 0.371 | 0.611 | 0.741 | 0.462 | 0.173 | 0.321 |
| RF | 0.698 | 0.510 | 0.195 | 0.820 | 0.282 | 0.137 | 0.233 |
| GBM | 0.696 | - | - | 0.818 | - | 0.143 | - |
| LR | 0.688 | 0.604 | 0.101 | 0.825 | 0.174 | 0.136 | 0.194 |
| LGBM | 0.796 | 0.453 | 0.542 | 0.798 | 0.494 | 0.140 | 0.371 |

* AUC, area under curve of the receiver operating characteristic curve.

| **Supplementary Table S3.** Performance of eight ML models for predicting mortality. | | | | | | | |
| --- | --- | --- | --- | --- | --- | --- | --- |
| Models | AUC | Precision | Recall | Accuracy | F1 score | Brier Score | Matthews  Correlation  Coefficient |
| **Training set** |  |  |  |  |  |  |  |
| SVM | 0.719 (0.696-0.741) | 0.500 (0.163-0.837) | 0.012 (0.003-0.020) | 0.961 (0.960-0.961) | 0.022 (0.007-0.038) | 0.038 (0.037-0.038) | 0.071 (0.024-0.117) |
| KNN | 0.558 (0.535-0.580) | 0.138 (0.071-0.205) | 0.027 (0.010-0.045) | 0.955 (0.954-0.956) | 0.045 (0.018-0.072) | 0.046 (0.045-0.048) | 0.045 (0.012-0.078) |
| NB | 0.867 (0.856-0.879) | 0.185 (0.170-0.201) | 0.692 (0.656-0.729) | 0.866 (0.854-0.878) | 0.292 (0.272-0.312) | 0.127 (0.116-0.137) | 0.311 (0.288-0.334) |
| MLP | 0.835 (0.811-0.859) | 0.386 (0.337-0.434) | 0.327 (0.257-0.396) | 0.951 (0.943-0.958) | 0.338 (0.292-0.384) | 0.038 (0.031-0.044) | 0.322 (0.285-0.359) |
| RF | 0.776 (0.760-0.792) | 0.528 (0.394-0.663) | 0.052 (0.036-0.067) | 0.961 (0.960-0.962) | 0.094 (0.067-0.120) | 0.035 (0.034-0.036) | 0.155 (0.113-0.197) |
| GBM | 0.853 (0.845-0.862) | - | - | 0.960 (0.960-0.961) | - | 0.035 (0.035-0.036) | 0.000 (0.002-0.001) |
| LR | 0.767 (0.746-0.788) | 0.372 (0.161-0.583) | 0.020 (0.009-0.031) | 0.960 (0.959-0.961) | 0.038 (0.017-0.058) | 0.036 (0.036-0.037) | 0.075 (0.034-0.117) |
| LGBM | 0.927 (0.921-0.934) | 0.355 (0.329-0.381) | 0.601 (0.561-0.642) | 0.941 (0.937-0.945) | 0.446 (0.417-0.475) | 0.044 (0.042-0.045) | 0.433 (0.402-0.465) |
| **Test set** |  |  |  |  |  |  |  |
| SVM | 0.751 | 0.500 | 0.006 | 0.961 | 0.012 | 0.037 | 0.051 |
| KNN | 0.590 | 0.381 | 0.047 | 0.960 | 0.083 | 0.042 | 0.123 |
| NB | 0.913 | 0.203 | 0.784 | 0.872 | 0.323 | 0.120 | 0.357 |
| MLP | 0.878 | 0.479 | 0.404 | 0.96 | 0.438 | 0.031 | 0.419 |
| RF | 0.820 | 0.722 | 0.076 | 0.963 | 0.138 | 0.033 | 0.227 |
| GBM | 0.889 | 1.000 | 0.006 | 0.961 | 0.012 | 0.034 | 0.075 |
| LR | 0.795 | 0.500 | 0.012 | 0.961 | 0.023 | 0.035 | 0.072 |
| LGBM | 0.940 | 0.403 | 0.696 | 0.948 | 0.511 | 0.040 | 0.506 |

* AUC, area under curve of the receiver operating characteristic curve.

| **Supplementary Table S4.** Performance of LGBM model for predicting AKI. | | | | | | | |
| --- | --- | --- | --- | --- | --- | --- | --- |
| Models | AUC | Precision | Recall | Accuracy | F1 score | Brier Score | Matthews  Correlation  Coefficient |
| **Training set** |  |  |  |  |  |  |  |
| Top 5 features | 0.747 (0.739-0.755) | 0.334 (0.327-0.341) | 0.653 (0.640-0.666) | 0.700 (0.694-0.706) | 0.442 (0.433-0.451) | 0.198 (0.195-0.200) | 0.293 (0.280-0.306) |
| Top 10 features | 0.767 (0.758-0.776) | 0.358 (0.346-0.370) | 0.667 (0.645-0.689) | 0.722 (0.713-0.731) | 0.466 (0.451-0.481) | 0.187 (0.185-0.190) | 0.327 (0.305-0.349) |
| Top 15 features | 0.780 (0.770-0.790) | 0.390 (0.378-0.403) | 0.656 (0.641-0.671) | 0.751 (0.742-0.759) | 0.489 (0.476-0.502) | 0.174 (0.171-0.177) | 0.358 (0.340-0.376) |
| Top 20 features | 0.787 (0.777-0.796) | 0.393 (0.381-0.404) | 0.654 (0.641-0.667) | 0.753 (0.745-0.761) | 0.491 (0.479-0.503) | 0.171 (0.167-0.175) | 0.360 (0.344-0.377) |
| All features | 0.806 (0.797-0.816) | 0.485 (0.470-0.499) | 0.559 (0.544-0.575) | 0.812 (0.806-0.817) | 0.519 (0.506-0.533) | 0.135 (0.132-0.138) | 0.404 (0.387-0.421) |
| **Test set** |  |  |  |  |  |  |  |
| Top 5 features | 0.744 | 0.333 | 0.660 | 0.698 | 0.443 | 0.197 | 0.295 |
| Top 10 features | 0.759 | 0.346 | 0.648 | 0.713 | 0.451 | 0.189 | 0.306 |
| Top 15 features | 0.767 | 0.364 | 0.640 | 0.732 | 0.464 | 0.182 | 0.324 |
| Top 20 features | 0.774 | 0.383 | 0.632 | 0.748 | 0.477 | 0.173 | 0.342 |
| All features | 0.796 | 0.453 | 0.542 | 0.798 | 0.434 | 0.140 | 0.372 |

* AUC, area under curve of the receiver operating characteristic curve.

| **Supplementary Table S5.** Performance of LGBM model for predicting mortality. | | | | | | | |
| --- | --- | --- | --- | --- | --- | --- | --- |
| Models | AUC | Precision | Recall | Accuracy | F1 score | Brier Score | Matthews  Correlation  Coefficient |
| **Training set** |  |  |  |  |  |  |  |
| Top 5 features | 0.870 (0.859-0.882) | 0.203 (0.196-0.211) | 0.611 (0.579-0.644) | 0.890 (0.885-0.895) | 0.305 (0.294-0.316) | 0.078 (0.076-0.080) | 0.309 (0.294-0.323) |
| Top 10 features | 0.895 (0.884-0.906) | 0.251 (0.234-0.267) | 0.587 (0.552-0.622) | 0.914 (0.909-0.919) | 0.351 (0.331-0.372) | 0.059 (0.057-0.060) | 0.346 (0.323-0.369) |
| Top 15 features | 0.913 (0.901-0.925) | 0.294 (0.277-0.312) | 0.632 (0.585-0.678) | 0.926 (0.922-0.929) | 0.401 (0.377-0.425) | 0.054 (0.052-0.056) | 0.398 (0.370-0.426) |
| Top 20 features | 0.919 (0.907-0.930) | 0.318 (0.295-0.341) | 0.644 (0.592-0.697) | 0.931 (0.927-0.936) | 0.425 (0.396-0.454) | 0.051 (0.049-0.053) | 0.421 (0.389-0.454) |
| All features | 0.927 (0.921-0.934) | 0.355 (0.329-0.381) | 0.601 (0.561-0.642) | 0.941 (0.937-0.945) | 0.446 (0.417-0.475) | 0.044 (0.042-0.045) | 0.433 (0.402-0.465) |
| **Test set** |  |  |  |  |  |  |  |
| Top 5 features | 0.885 | 0.206 | 0.591 | 0.895 | 0.305 | 0.074 | 0.306 |
| Top 10 features | 0.908 | 0.264 | 0.596 | 0.920 | 0.366 | 0.057 | 0.361 |
| Top 15 features | 0.927 | 0.335 | 0.731 | 0.933 | 0.460 | 0.050 | 0.467 |
| Top 20 features | 0.929 | 0.351 | 0.702 | 0.938 | 0.468 | 0.048 | 0.469 |
| All features | 0.940 | 0.403 | 0.696 | 0.948 | 0.511 | 0.040 | 0.506 |

* AUC, area under curve of the receiver operating characteristic curve.
